# Utilizing gold nanoparticles in plasmonic photothermal therapy for cancer treatment

**DOI:** 10.1016/j.heliyon.2025.e42738

**Published:** 2025-02-15

**Authors:** Amina Badir, Siham Refki, Zouheir Sekkat

**Affiliations:** a Department of Chemistry, Faculty of Sciences, Mohammed V University, Rabat, Morocco; b Optics and Photonics Center, Moroccan Foundation for Advanced Science Innovation and Research, MAScIR, University Mohammed VI Polytechnic, Benguerir, Morocco

**Keywords:** Gold nanoparticles, Cancer, Photothermal therapy, Localized surface plasmon resonance, Surface functionalization, Laser

## Abstract

In recent decades, significant attention has been directed towards gold nanoparticles due to their exceptional properties, capturing the interest of researchers globally. Their unique characteristics, such as localized surface plasmon resonance, high surface area to volume ratio, biocompatibility, and facile surface functionalization, render them highly suitable for diverse applications, ranging from optoelectronics and sensing to surface-enhanced spectroscopies and biomedical uses, particularly in the realm of photothermal therapy. Plasmonic photothermal therapy, an emerging biomedical technology, has garnered substantial interest for its potential in cancer treatment and management. This approach employs photothermal agents, such as gold nanoparticles, which absorb light in the near-infrared region. When these agents accumulate within cancer cells, the absorbed photon energy is converted into heat, inducing local hyperthermia. This localized effect selectively eliminates damaged cells adjacent to nanoparticles while sparing normal cells. Various shapes and sizes of gold nanoparticles have proven well-suited candidates for photothermal therapy. This paper provides an overview of the distinctive properties of gold nanoparticles. It delves into the surface functionalization techniques crucial for ensuring cancer cells' effective retention and targeting of gold nanoparticles. In this context, the present paper reviews diverse applications of gold nanoparticles with different shapes in plasmonic photothermal therapy, encompassing nanospheres, nanorods, nanoshells, nanostars, and nanocages.

## 1 Introduction

Cancer serves as a general descriptor for a diverse spectrum of illnesses where malignant cells undergo unrestrained and excessive proliferation, potentially impacting nearly any organ or tissue within the body. According to the World Health Organization (WHO), globally, cancer is the second leading cause of death; it is responsible for one out of every six deaths worldwide. In a joint report, the World Cancer Research Fund (WCRF) and the American Institute of Cancer Research (AICR) stated that 19.3 million new cancer cases and almost 10.0 million cancer deaths were estimated in 2020 around the world [1]. Acknowledged as a significant public health concern, the Global Cancer Observatory (GCO) predicts a substantial increase in the global burden of cancer, reaching an estimated 27.5 million cases by the conclusion of 2040, primarily attributed to population growth. The intricate nature of cancer poses a challenge in treatment, given its dynamic and evolving characteristics over time, marked by the accumulation of new mutations [2].

Nevertheless, progress in the field of cancer biology has significantly contributed to the emergence of novel methodologies in cancer treatment [3]. Conventional treatment modalities involving surgical interventions, therapies such as radiotherapy and chemotherapy, and various combinations thereof stand as the predominant approaches employed in the treatment of cancer. Surgery is the oldest oncological discipline; it effectively removes early-stage solid tumors that are localized in a specific area and have not yet migrated to other parts of the body. Surgery is supposed to be a therapeutic option for cancer removal and tumor mass reduction. However, researchers revealed that the surgical approach may not be universally applicable across all stages of cancer. It was evidenced that surgery can potentially facilitate the metastatic dissemination of tumor cells. In simpler terms, metastatic cells can evade the primary tumor at its initial stages, giving rise to clinically undetected micro metastases in different body regions [4].

Surgical removal induces local and systemic inflammatory reactions while constraining the immunological response. Radiation therapy, or radiotherapy, constitutes a fundamental cancer treatment deemed necessary in over 60 % of individuals diagnosed with cancer [5]. Radiotherapy is a targeted cancer treatment that uses radiation to eliminate cancer cells by damaging their DNA, thus inhibiting their growth and progression. Various types of radiation can be employed in this therapy, including X-rays, gamma rays, and photon beams. Radiotherapy can be categorized primarily as external beam radiotherapy (EBRT), involving the administration of radiation beams externally, and internal beam radiotherapy (IBRT), wherein the radiation is delivered within the body [6]. While radiotherapy is commonly utilized in cancer treatment, it has the potential to harm normal cells, resulting in various side effects, including fatigue, nausea, swallowing, soreness, hair loss, skin irritation, and tooth decay [5]. Despite its drawbacks, surgery and radiotherapy remain the most efficient traditional therapies for local tumors.

Nevertheless, the efficacy of these treatments diminishes when cancer disseminates throughout the body. Therefore, chemotherapy is employed for metastatic cancers. Chemotherapy is the use of drugs to destroy malignant tumors. It works by preventing cancer cells from growing, dividing, and reproducing. It can get to each organ in the body over blood circulation [7]. Anti-cancer drugs are based on toxic compounds which limit the rapid proliferation of cancer cells; however, some normal cells are fast-growing too, such as hair follicles, bone marrow, and gastrointestinal tract cells, therefore they can be affected by these drugs, and this leads to the harmful side effects seen after the treatment [8]. Chemotherapy is associated with a range of adverse effects that have the potential to harm various organs in the body, including vital ones such as the heart, brain, and lungs.

**Fig. 1:**
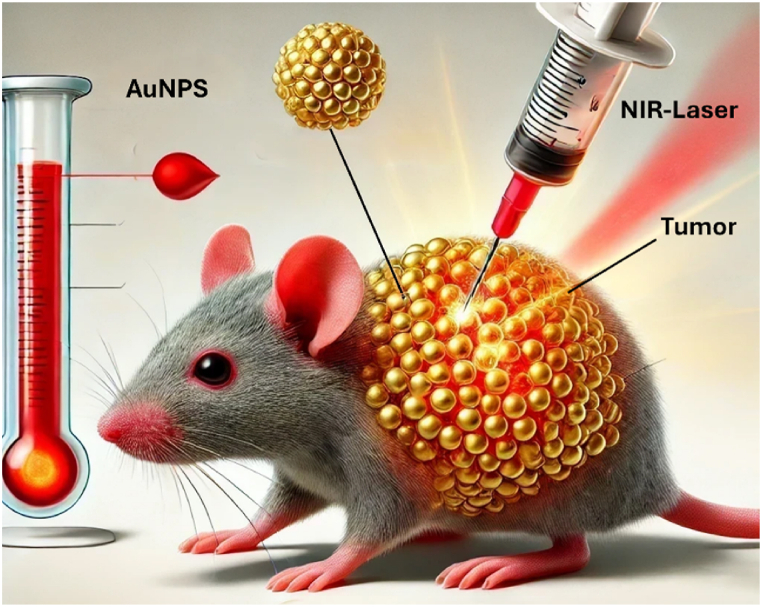
Schematic illustration of gold nanoparticles used in photothermal therapy within a mouse body. The diagram illustrates a mouse injected with gold nanoparticles in the treatment area. A NIR Laser is applied to irradiate the nanoparticles, inducing localized heating. A thermometer is depicted near the treatment area to indicate the temperature changes from nanoparticle irradiation. This thermal energy induces targeted hyperthermia, selectively destroying cancerous cells while minimizing damage to surrounding healthy tissues.

Traditional cancer treatment methods, including chemotherapy, radiotherapy, and surgery, are fundamental and effective strategies in cancer management, yet each is associated with notable challenges. Chemotherapy, while effective for systemic and metastatic cancers, often leads to systemic toxicity, drug resistance, and compromised immune function [9,10]. Radiotherapy provides precise targeting and high local tumor control, particularly with advanced technologies, but it remains limited to localized cancers and carries the risk of long-term complications [11]. Surgery offers the potential for complete tumor removal and immediate symptom relief, though it is invasive and less effective for metastatic disease. Despite their effectiveness, these methods frequently cause significant damage to healthy tissues, resulting in severe side effects. Optimizing treatment outcomes often requires the strategic combination of these approaches, tailored to the specific cancer type, stage, and patient condition [10]. These limitations highlight the need for alternative therapies with greater precision and fewer adverse effects. A promising breakthrough in this context is photothermal therapy (PTT), which has gained significant attention in cancer treatment research [12]. PTT works by leveraging the ability of nanoparticles to convert absorbed laser light into localized heat, effectively destroying cancer cells in the targeted area. This heat generation mechanism ensures the selective elimination of malignant cells and minimizes damage to surrounding healthy tissues, making PTT a minimally invasive and highly targeted treatment [13,14]. Unlike chemotherapy, which relies on systemic drug delivery and affects both healthy and cancerous cells, PTT achieves precise targeting through the use of photothermal agents (PTAs), which selectively accumulate in tumor cells [15]. Similarly, PTT reduces the risks associated with radiotherapy [16], such as collateral damage to surrounding healthy tissues, by employing near-infrared (NIR) light to generate localized heat in tumor areas. This mechanism spares adjacent healthy tissues and avoids the chemical and invasive processes characteristic of traditional therapies. Additionally, PTT's versatility extends to its applicability across various tumor types and its potential to integrate with other treatments, such as immunotherapy and surgery, for enhanced outcomes [17]. PTT relies on the accumulation of photothermal agents (PTAs) in cancer cells, accompanied by light irradiation of the targeted cell [18]. These photosensitizers can absorb light and turn it into heat when they are stimulated by electromagnetic radiation, generally NIR light, which raises the surrounding environment's temperature and induces the destruction of cancer cells that are tolerant to heat (Fig. 1).

It has been reported that cancer cells can successfully be eliminated when the temperature of the tumor location exceeds 42 °C [19]. Tumors are selectively targeted for destruction to prevent any impact on neighboring normal cells, thereby minimizing potential side effects. Selecting the appropriate PTAs is vital for achieving optimal therapeutic outcomes. These agents may encompass dyes, nanoparticles, or any other materials capable of absorbing light. Additionally, PTAs should acquire strong light absorption, particularly in the near-infrared region, as they can penetrate deeply into tissues and traverse biological materials like skin with low absorption and scattering. They should also demonstrate high photothermal conversion efficiency and excellent biocompatibility [20]. Recent advances in nanotechnology have enabled the development of various nanomaterials that possess excellent efficiency for different biomedical applications [21] and outstanding photothermal conversion ability [22]. These nanomaterials have the potential to serve as highly effective PTAs. They can be categorized into two groups: organic PTAs, which encompass dyes (such as cyanine, croconaine, and porphyrin), polymers (including polyaniline, polypyrrole), and chitosan, and inorganic PTAs, comprising nanostructures (gold, palladium, copper), carbon-based materials, and quantum dots [23].

Due to their distinctive optical characteristics, the prevalent choice for photosensitizers in PTT often revolves around using gold nanoparticles. The unique optical properties of gold nanoparticles (AuNPs) make them a widely favored option for PTT applications. AuNPs with diverse shapes manifest unique localized surface plasmon resonance (LSPR) peak positions, a characteristic influenced by both the size and shape of the nanoparticles. This distinct attribute enhances their capacity to efficiently convert light into heat efficiently [24]. Upon exposure to NIR light, gold nanoparticles undergo a process where the absorbed light energy induces an oscillation of the plasmonic electrons on their surface at a heightened frequency. This phenomenon generates heat, a thermal effect that can eliminate cancer cells [25]. Various gold nanoparticle configurations have been scrutinized for their potential application in cancer photothermal therapy. These configurations encompass gold nanospheres, nanorods, nanostars, nanoshells, and nanocages. Each distinct shape possesses characteristic features that make it suitable for deployment as a photothermal agent. This paper provides an in-depth review of recent progress in utilizing gold nanostructures for photothermal therapy. We start with a comprehensive overview of nanoparticle properties, emphasizing their exceptional photothermal efficiency in the context of cancer treatment. A discussion ensues regarding the significance of surface functionalization of nanoparticles and various techniques employed for this purpose. Subsequently, we delve into the application of diverse shapes of gold nanoparticles in the realm of cancer PTT, spotlighting recent contributions within this domain. Additionally, we touch upon various factors influencing the effectiveness of PTT in the context of cancer. Furthermore, we address the limitations and challenges associated with using AuNPs, including issues related to biocompatibility, biodistribution, clearance, and scalability. By integrating these discussions, this work aims to guide researchers in developing effective strategies for the clinical implementation of PTT.

## 2 Gold nanoparticles and their application in PTT

### 2.1 Properties of gold nanoparticles

**Fig. 2:**
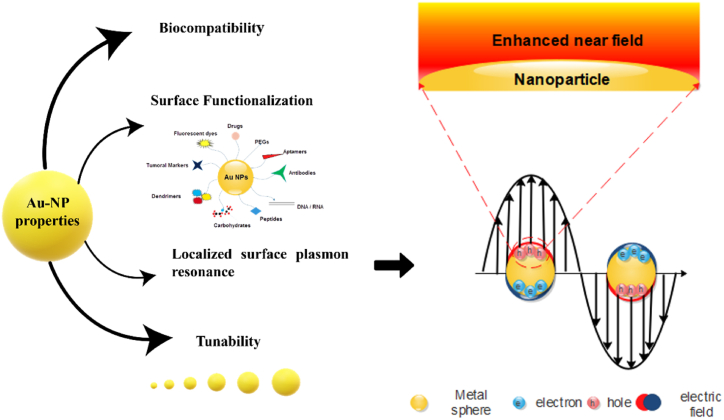
Schematic illustration depicting the key properties of gold nanoparticles. The Figure highlights various characteristics, including their surface chemistry, biocompatibility, and optical properties. It also illustrates the phenomenon of electric field enhancement, demonstrating how gold nanoparticles amplify local electric fields due to surface plasmon resonance. This enhanced electric field contributes to their unique behavior and applications, such as improved light absorption, surface functionalization capabilities, and versatility in biomedical and technological fields.

In the realm of photothermal applications, gold nanoparticles (AuNPs) have emerged as highly promising photothermal agents (PTAs) due to their distinctive physical, chemical, and optical properties [26]. Their strong light absorption and rapid heat conversion capabilities are attributed to LSPR [27]. LSPR is the phenomenon where the electromagnetic field induces a coherent oscillation of the metal's conduction band electrons, resulting in a bipolar oscillation along the electric field upon illumination (Fig. 2); this boosts absorption and scattering properties [25]. The position and intensity of the surface plasmon resonance (SPR) band depend on factors such as nanoparticle size, shape, metal type, and surface composition [28].

Gold nanoparticles exhibit a high surface-to-volume ratio [29], facilitating the conjugation of numerous biomolecules to their surface, thus enhancing their targetability and specificity for cancer therapy [30]. For photothermal therapy, researchers focus on nanoparticles with specific sizes and shapes that exhibit strong absorption in the near-infrared region [31]; this is crucial because physiological fluids have minimal absorption in this range, allowing deep tissue penetration. AuNPs are stable, have low reactivity, and can be used safely in biological systems without significant toxicity. Their biocompatibility has been evaluated using various in vivo and in vitro models and can be improved through surface coating [32]. AuNPs can bind with thiol groups, permitting their attachment to various organic ligands or polymers with specific functions [33]. They can efficiently absorb light and convert it into heat, which can be used to destroy cancer cells [34]. Tumors, characterized by abnormal and often leaky vasculature, are easier to penetrate with small AuNPs, enabling them to reach cancer cells effectively. The efficacy of gold nanoparticles as PTAs can be further understood through theoretical modeling. Smaller nanoparticles have a larger surface area-to-volume ratio, which improves their capacity for effective light absorption. At smaller scales, their interactions with light create stronger resonances, especially in the near-infrared range, where many biological tissues are transparent. As a result, absorption predominates over scattering, enhancing photothermal conversion efficiency. Thus, higher photothermal efficiency is attained with smaller nanoparticles, as they primarily absorb light rather than scatter it [35].

### 2.2 Surface modification of AuNPs

As previously mentioned, gold nanoparticles offer several advantages compared to other photothermal agents. However, the risk of long-term toxicity can be one of the most challenging aspects of in vivo photothermal therapy. Therefore, nanoparticle surface modification is crucial to improve their biocompatibility, reduce their cytotoxicity, increase their stability, and achieve specific target delivery [36]. Surface modification is a helpful technique that aims to improve the properties of gold nanoparticles so that they can be adapted for different medicinal applications. Small proteins, peptides, antibodies, aptamers, and oligosaccharides are all examples of molecules applied to change the AuNP's surface [37]. The selection of functionalization techniques for gold nanoparticles in photothermal cancer therapy is guided by the need to balance stability, biocompatibility, and targeting specificity. Proteins such as Bovine serum albumin (BSA) are one of the common ligands used to conjugate gold nanoparticles for photothermal cancer therapy [38]. They are known for their high specificity, biocompatibility, and stability**.** A study [39] highlights that precoating nanoparticles with BSA can enhance their biocompatibility and decrease leukocyte association. This modification offers a natural biomolecular interface, which helps reduce immunogenicity and improve targeting efficiency, making it a promising approach in nanomedicine. Functionalization with polymer coatings like polyethylene glycol (PEG) enhances nanoparticle stability, biocompatibility, and reduces nonspecific interactions. They facilitate surface modification, improve colloidal stability, and enable effective cargo loading [40,41]. Immunoglobulin G (IgG), and Transferrin [37], have been used in conjugation with nanoparticles to target specific antigens expressed on cancer cells by enabling specific binding to tumor antigens [42]. Another option for the selective targeting of gold nanoparticles in cancer cells is aptamers. Aptamers are short, single-stranded nucleic acids that bind to specific targets with high affinity, excellent stability, and low immunogenicity [43]. Many studies have demonstrated using aptamers such as AS42 [44] and AS1411 [45], which can bind to proteins overexpressed in cancer cells. This property enables their use as targeting moieties for delivering therapeutic agents in cancer gene therapy, enhancing treatment specificity and efficacy [46]. Moreover, peptides offer several advantages, such as targeting ligands for nanoparticle-based photothermal therapy. They have high binding affinity and selectivity for specific receptors on cancer cells and can be easily conjugated to nanoparticles [47]. Lipid-based modifications, such as liposome encapsulation, significantly enhance nanoparticle delivery and cellular uptake. These lipid nanoparticles (LNPs) leverage the biocompatibility and biodegradability of lipids to improve drug solubility, achieve controlled release, and facilitate targeted delivery, effectively overcoming biological barriers like the blood-brain barrier [48]. Lastly, small molecule ligands like folic acid play a crucial role in enhancing the selectivity of drug delivery systems for cancer cells through receptor-mediated targeting. Folic acid is particularly effective due to its ability to bind to folate receptors, which are often overexpressed in various tumors. This specificity not only improves the therapeutic efficacy of anticancer agents but also minimizes side effects associated with conventional therapies [49]. Each approach has distinct advantages, and the choice depends on the specific requirements of the intended type of cancer being targeted. Table 1 provides a comprehensive overview of various surface functionalization methods applied to gold nanoparticles for their use in photothermal therapy. It highlights the functionalization agents, their mechanisms of action, and their specific advantages in enhancing the efficacy, targeting, and biocompatibility of AuNP-based therapeutic systems.

**Table 1:** Overview of surface functionalization methods for gold nanoparticles in photothermal therapy.

Modification Technique	Example of Fonctionnalisation Agent	Advantages of Photothermal Therapy	Reference
Polymer Coating	Polyethylene glycol (PEG)	Improved biocompatibility and extended circulation time	[50,51]
Proteins Coating	Bovine Serum Albumin	Enhanced cellular uptake and specific targeting capabilities	[38,52]
Antibodies Functionalization	Immunoglobulin G (IgG)	Provides high specificity for recognizing cancer-specific biomarkers	[53,54]
Aptamer Fonctionnalisation	DNA or RNA Aptamers	Exhibits strong binding affinity and minimal immunogenic response	[44,55]
Lipids-Based Modification	Liposomes	Enhances particle stability and enables targeted drug delivery	[56,57]
Small Molecule Ligands	Folic acid	Facilitates targeted delivery to folate receptor-positive cancer cells and allows for straightforward conjugation	[58,59]

**Fig. 3:**
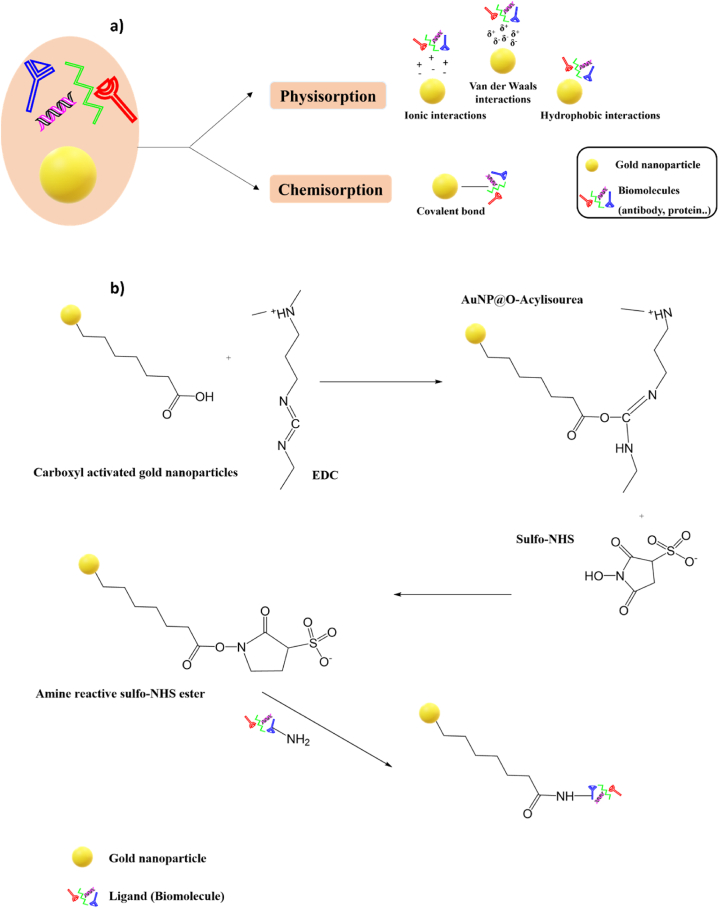
Gold nanoparticles conjugation techniques: **a)** Different techniques of gold nanoparticles conjugation with biomolecules, **b)** Example of covalent binding of gold nanoparticles.

Gold nanoparticle conjugation with biomolecules could be achieved through two different mechanisms: chemisorption and physisorption (Fig. 3-a). Chemisorption (or covalent binding) involves the formation of chemical bonds between the nanoparticle and the biomolecule, whereas physisorption (non-covalent binding) implies electrostatic, hydrogen, hydrophobic, and van der Waals interactions [60]. The non-covalent approach is defined as the spontaneous absorption of biomolecules onto the surface of the nanoparticles; it might be achieved through hydrophobic interactions, ionic interactions, etc. [61]. It is the most straightforward conjugation method. For example, hydrophobic interactions occur when the hydrophobic part of the biomolecule attracts the surface of the metal nanoparticle and forms a non-covalent bond. Furthermore, biomolecules, especially antibodies, have excess positively charged groups, such as amino acids, allowing ionic interactions to bind with the surface of the negatively charged nanoparticle [62]. For example, some researchers conjugated gold nanoparticles to 5-aminolevulinic acid for photodynamic therapy application through electrostatic interactions [63]. In another study, hypericin was physically adsorbed onto gold nanoparticles to enhance the therapy of MCF-7 breast cancer cells [64]. Despite its ease, physisorption may have some weaknesses, including the random orientation of the antibodies at the gold nanoparticle and the weak attachment, which means other molecules in the sample can efficiently serve as a suitable substitute [65]. The covalent approach forms a stable chemical bond between the nanoparticle and the biomolecule. It requires the chemical modification of either the nanoparticle or the biomolecule or sometimes both. It results in a stable conjugate in which the biomolecule is tightly bound to the nanoparticle's surface [66]. One common method for covalent conjugation via chemical modification of gold nanoparticles is by adding an organic functional group (R-NH2, R-COOH, …etc.) on the nanoparticle's surface, which may bind biological molecules. For noble metals, such as gold, nanoparticles are functionalized with crosslinkers having -SH or -NH_2_ groups on one edge and another functional group on the other edge to facilitate the binding of biological ligands [32]**.** One example of the crosslinkers used are polymers such as thiol polyethylene glycol with a carboxylic acid (SH-PEG-COOH), which can covalently bind to the nanoparticle. It is characterized by low toxicity, low immunological response, and high water solubility and stability [67]. 1-Ethyl-3-(3-dimethylaminopropyl) carbodiimide (EDC) and N-hydroxysulfosuccinimide (NHS) are used to activate carboxylic groups, forming an unstable intermediate molecule that easily interacts with primary amines (Fig. 3-b). This main amine of the biomolecule will create an amide bond with the initial -COOH, allowing to form a covalent bond with the nanoparticle [68]**.** In general, sulfhydryl groups can generate a strong Au-S bond, which makes them extremely appealing for the conjugation of gold nanoparticles with biomolecules such as antibodies. So, the covalent conjugation could also be achieved by modifying the surface of the antibody. Typically, it involves introducing sulfhydryl groups on the antibody's surface that can then react with the surface of the AuNP. These functional groups could be generated via either the reaction with primary amines or by the reduction of native disulfide bonds of antibodies [69]. Several research studies have used covalent binding to attach gold nanoparticles and biomolecules. For example, folic acid was covalently bonded to the surface of gold nanorods with a silane coupling agent. The obtained folic acid-conjugated silica-modified AuNRs demonstrate very selective targeting, improved radiation treatment, and photothermal therapy [70]. Other researchers used chemisorption to conjugate monoclonal antibodies to gold nanostars for plasmonic photothermal therapy of gastric cancer cells [71]. Another study reported that the CD133 antibody was covalently bound to gold nanostars for combined therapy to treat prostate cancer [72]. Furthermore, it was reported that arginine-glycine-aspartic acid was covalently conjugated to gold nanostars to serve as an efficient photothermal agent for cancer therapy [73]. Other conjugation strategies require the modification of the surface of the gold nanoparticle as well as the surface of the antibody in order to build a strong, stable bond, and highly specific interactions between the AuNP and the biomolecule [74].

## 3 Laser irradiation and interaction mechanism

Laser irradiation is one of the crucial processes in photothermal therapy. As mentioned, NIR lasers are commonly employed because they efficiently heat nanoparticles deep within the body and their exceptional tissue penetration capabilities. NIR Photothermal treatment may be conducted using two different laser settings: pulse mode and continuous mode (CW). A pulsed laser generates high-intensity light pulses with regulated durations. It provides precise control over the thermal radiation, enabling tissue-specific targeting with minimal harm to healthy cells. The continuous wave laser delivers a continuous beam of light, allowing the gold nanoparticles to generate constant heat to induce hyperthermia and ultimately eliminate the targeted cells. When the laser power is minimal, it is exposed for an extended period, when laser intensity is significant, tissue exposure time is decreased [75]. The specific irradiation wavelength depends on the photothermal agent used; gold nanoparticles exhibit strong absorption in the NIR range, typically between 650 nm and 900 nm; therefore, lasers within this range are required for an efficient photothermal effect.

**Fig. 4:**
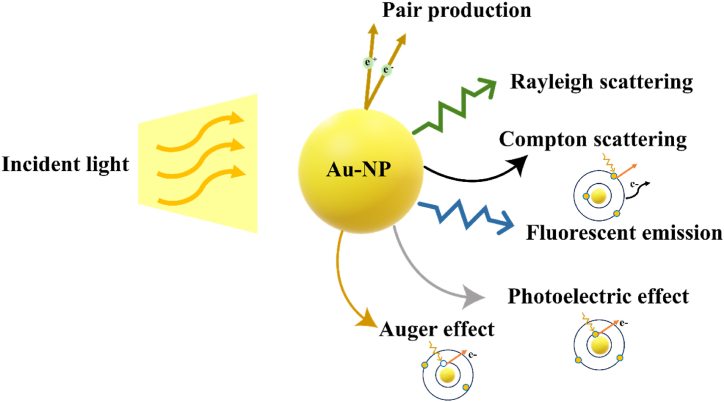
Schematic representation of potential interactions between an incident photon and a Gold Nanoparticle. This figure illustrates the interactions between an incident photon and a gold nanoparticle. The interactions include Rayleigh scattering, Compton scattering, fluorescence emission, the photoelectric effect, the Auger effect, and pair production.

The interaction of gold nanoparticles with laser light in photothermal therapy for cancer involves various principles. Initially, it is important to consider the potential interactions between an incident photon and a gold nanoparticle (Fig. 4). These interactions include Rayleigh scattering, Compton scattering, fluorescence emission, the photoelectric effect, the Auger effect, and pair production. Each pathway represents a distinct process through which photons interact with the nanoparticle, influencing its optical and electronic properties. A key aspect of this interaction is localized surface plasmon resonance (LSPR), which occurs when electrons in the nanoparticle's metal structure collectively oscillate in response to incident light. Consequently, when nanoparticles interact with laser light, the light can be absorbed or scattered. Plasmon resonance primarily dictates absorption, allowing the nanoparticle to absorb light at its resonant wavelength. Depending on the energy transfer with the nanoparticle, the dispersed light may be inelastic (Raman scattering) or elastic (Rayleigh scattering) [76]. Next, once the nanoparticles absorb laser light, they transform the light's energy into heat energy via a phenomenon known as photothermal conversion. The captured light energy triggers the nanoparticles' plasmons, immediately releasing energy in heat. After the nanoparticles generate heat, the heat transfer can elicit hyperthermia, thereby increasing the tumor's temperature to a level that damages cancer cells. In other words, the interaction between laser light and nanoparticles results in the stimulation of electrons within the nanoparticles, subsequently leading to electron relaxation. The relaxation of electrons induces an increase in kinetic energy, leading to heat generation. Nanoparticles possess the ability to conduct heat within cancerous tissue, thereby leading to the eradication of these cancerous cells [77].

## 4 The photothermal effect of different shapes of gold nanoparticles

Gold nanostructures, including nanospheres, nanorods, nanoshells, nanostars, and nanocages, exhibit diverse morphologies, making them highly promising photothermal agents. These structures offer distinct properties that are particularly advantageous for applications in cancer therapy [78]. For example, gold nanospheres (AuNSps) are characterized by their ease of synthesis and functionalization [79]. Gold nanorods (AuNRs) exhibit two surface plasmon resonance peaks, corresponding to the transversal and longitudinal (which absorb in the NIR range) SPR effects [80]; therefore, when exposed to NIR light, AuNRs may effectively convert light into heat through the SPR effect [81,82]. Their large surface area distinguishes gold nanostars (AuNSts) owing to their multiple branches, which provide space for more plasma to absorb NIR light [83]. Gold nanoshells (AuNShs) have a core-shell structure of a silica core covered by a thin gold metallic shell. One of the most intriguing aspects of AuNShs is their unique surface plasmon resonance property, which can be finely tuned from the visible to NIR region [84]. Ultrathin porous walls and hollow interiors characterize gold nanocages (AuNCs), precisely tuned SPR bands, and strong absorption [85]. Table 2 comprehensively analyzes the gold nanoparticle shapes commonly employed in PTT, highlighting their unique physicochemical properties and associated limitations. The discussion emphasizes the advantages of these nanostructures, such as their tunable plasmonic characteristics, while addressing challenges, including synthesis complexities and biocompatibility issues. Building on this foundational understanding, the following section will explore case studies demonstrating the application of different gold nanoparticle morphologies in PTT, offering insights into their practical performance and therapeutic potential.

**Table 2:** Common shapes of gold nanoparticles used for PTT.

AuNP	Structure	Features	Limitations	Reference
Nanospheres		Ease of synthesis and surface functionalization Enhanced biocompatibility	Low absorption in the NIR region	[86]
Nanorods		Two SPR peaks: Strong absorption in the NIR region	Synthesis complexity: Requirement of cytotoxic reagents	[81]
Nanoshells		Core-shell structure: Fine-tuned from visible to NIR region.	Size and shell thickness control	[87]
Nanostars		Multiple branches: Large surface area	Larger hydrodynamic diameter: May reduce cellular uptake	[88]
Nanocages		Ultrathin porous walls and hollow interiors	Synthesis complexity	[89]

### 4.1 Au nanospheres

**Fig. 5:**
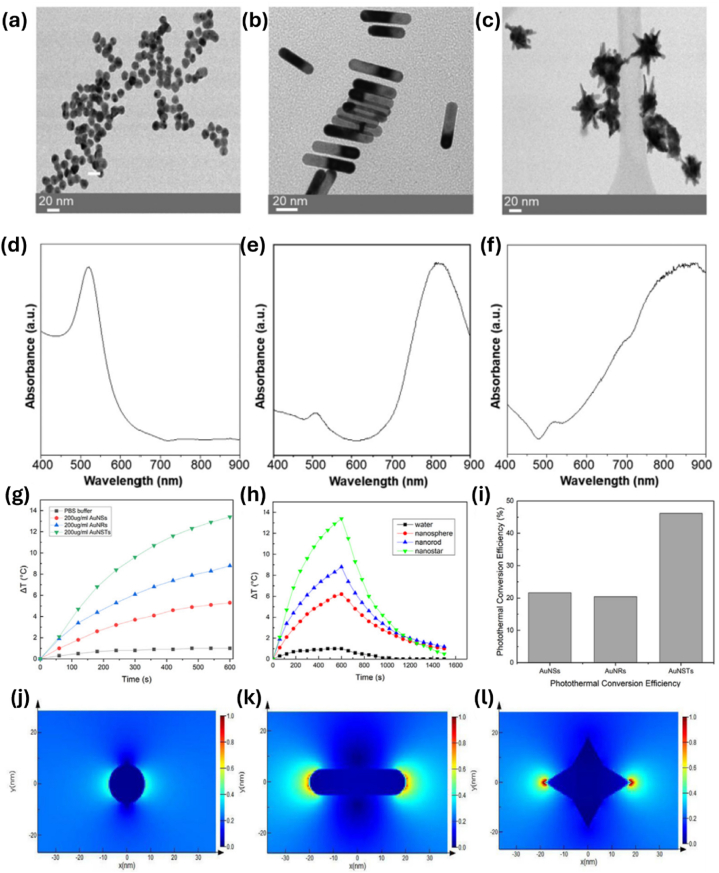
Characterization of gold nanoparticles with different morphologies and their photothermal conversion. Transmission electron microscopy (TEM) images of (a) AuNSs, (b) AuNRs, (c) AuNSTs; The normalized UV–Visible spectra of (d) AuNSps, (e) AuNRs, (f) AuNSTs. (g) under 808 nm NIR light (300 mW/cm2) triggered a temperature increase of AuNPs over 10 min. (h) Photothermal effects of the irradiation of the AuNPs in PBS buffer (200 mg/ml) with the laser irradiation, where the Laser was first irradiated for 600 s and then removed. (i) The photothermal conversion efficiency of the AuNPs in PBS buffer at a concentration of 200 mg/ml. FDTD of the electric field distribution of the gold nanoparticles with different morphologies under an 808 nm laser irradiation. (j) AuNSps, (k) AuNRs, (l) AuNSts. This figure has been reproduced from Ref. [78] with permission from Elsevier.

Gold nanospheres are the most easily obtained gold nanoparticles. The conventional Turkevich approach is the most widely used technique for synthesizing and stabilizing AuNSps in an aqueous phase [90]. It involves the reduction of gold salt (chloroauric acid, HAuCl_4_) with a reducing agent, generally sodium citrate, which acts as a reducing agent and a stabilizer. By changing the reagents' concentration, the nanospheres' size may vary from 1 nm to 100 nm. AuNSps show lower cytotoxicity, better biocompatibility, and easy functionalization, and they may smoothly be eliminated from the body [91]. Gold nanospheres' photothermal conversion efficiency was examined and reported by many researchers. For example, the efficiency of light-to-heat energy transfer was measured by exposing AuNSps ranging in size from 5 to 50 nm to a continuous-wave (CW) green Laser (532 nm). The photothermal conversion efficiency was discovered to be size-dependent; the efficiency increased as the size decreased [92]. In further investigation, AuNSs coupled with anti-epithelial growth factor receptor (EGFR) antibodies were shown to cause specific harm to cancer cells. Since oral squamous carcinoma cells overexpress the EGFR biomarker, antibody conjugation can boost the targeted delivery of nanoparticles to tumor cells [93]. A comparative study also investigated the photothermal performance of gold nanoparticles with different morphologies, including nanospheres, nanorods, and nanostars, by analyzing their UV–Vis absorption spectra, morphology by scanning electron microscopy, temperature evolution under 808 nm NIR laser irradiation, photothermal conversion efficiency, and theoretical calculations by FDTD (Fig. 5(a–l)). Gold nanospheres were surface modified with PEG, and their photothermal potential was evaluated by irradiating with an 808 nm NIR laser at a power density of 0.3 W/cm^2^. The results showed that AuNSps were only efficient by 21.6 % (Fig. 5-i) compared to other shapes. Gold nanospheres exhibit lower photothermal conversion efficiency than other nanostructures primarily due to their limited ability to resonate with near-infrared light directly. Instead, they rely on two-photon absorption and second-harmonic generation to absorb and convert light into heat [78]. A recent study showed that loading doxorubicin (DOX), an anticancer drug, onto gold nanospheres could efficiently treat breast cancer cells (MCF-7). Briefly, they synthesized small gold nanospheres conjugated with DOX, incubated them with MCF-7, and irradiated them with a 532 nm laser at a power density of 0.25 W/cm^2^. The findings showed that DOX@AuNPs increased anti-proliferation activity on cancer cells while decreasing cell viability [94]. However, gold nanospheres’ absorption is limited to the visible region, with SPR absorption peaks around 500 nm–600 nm, where the photons have poor tissue penetration depth and are challenging for in vivo photothermal applications, which are ideally performed in the NIR. Gold nanospheres could be exploited using aggregated or assembled systems to overcome these limitations. For example, gold nanoparticle clusters were developed as an effective NIR photothermal agent. Gold nanospheres were first synthesized and then assembled into clusters using cationic surfactants, and the photothermal effect was demonstrated using an 808 nm laser. With a maximum efficiency of 65 %, the results revealed a highly effective photothermal conversion of gold nanoparticle clusters. In order to maintain their biocompatibility and colloidal stability, gold nanoparticle clusters were coated with a silica layer [95]. Another study developed unique pimpled gold nanospheres and functionalized them with thiolate polyethylene glycol (SH-PEG400-SH). The light-to-heat conversion efficiency was investigated using an 808 nm laser at 4W/cm^2^. The findings show that pimpled gold nanospheres under laser irradiation could successfully damage cancer cells [96]. Another study demonstrated the effectiveness of melanin-gold hybrid nanoparticles as promising photothermal agents for targeted tumor treatment. These hybrid nanospheres exhibited enhanced near-infrared absorption and superior photothermal conversion compared to conventional nanoparticles. Surface functionalization with targeting ligands facilitated efficient cellular uptake and tumor localization in vivo, highlighting the importance of photothermal efficiency and precise delivery to tumor sites. This innovation underscores the potential of hybrid gold nanospheres for advancing photothermal cancer therapies [89].

### 4.2 Au nanorods

**Fig. 6:**
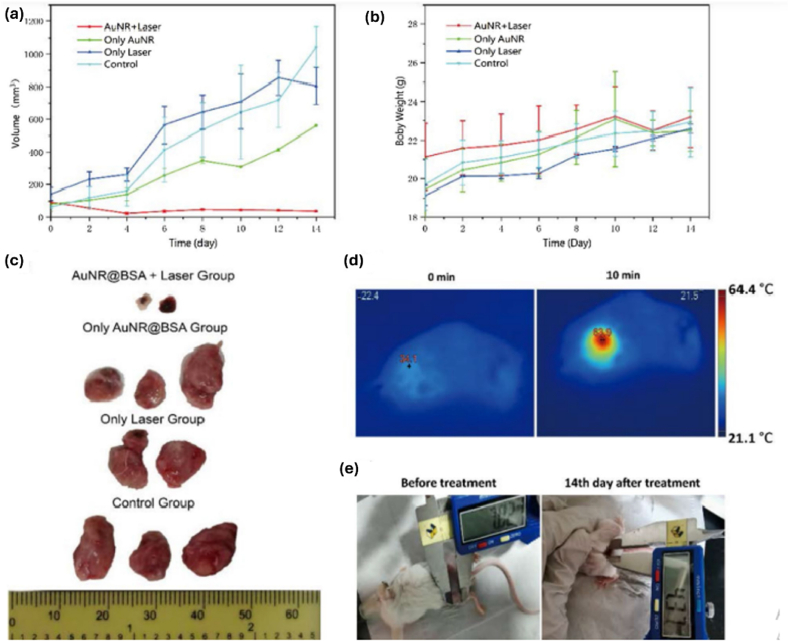
Photothermal treatment of tumor-bearing mice. (a) Tumor volume as a function of time under different treatment conditions. (b) Body weight changes of mice bearing tumors as a function of time, post-treatment. (c) Photographs of tumor tissues in different groups after 14-day treatment. (d) Infrared thermographic images of tumor-bearing mice exposed to a 1064 nm laser (0.75 W/cm2, 10 min) at 10 min post-intratumoral injection of AuNR@BSA. (e) Images of mice from AuNR@BSA_laser group after treatment. (mean ± SD, n = 2). This figure has been reproduced from Ref. [38] under the Creative Commons Attribution 4.0 International License (CC BY 4.0). Link to license: https://creativecommons.org/licenses/by/4.0/.

Gold nanorods are widely recognized for their strong absorption in the NIR region. They are also characterized by their ease of functionalization, great biocompatibility and targeting, and high photothermal conversion efficiency [97], which makes them excellent photothermal agents for in vitro and in vivo applications. AuNRs are generally prepared using the seed growth method [98], which includes first, the preparation of small gold seeds by reducing chloroauric acid (HAuCl_4_) with sodium borohydride in aqueous cetyltrimethylammonium bromide (CTAB) solution. Then, the freshly prepared seed solution is added to the growth solution, which contains reducing agents such as ascorbic acid and silver nitrate. The seed-mediated growth approach exhibits monodisperse Au nanorods with extremely high yields and good homogeneity. However, using the surfactant CTAB in synthesizing gold nanorods as a stabilizer remains cytotoxic. Therefore, a surface modification is well needed to increase the biocompatibility of AuNRs, and it was demonstrated that coating organic or inorganic materials on gold nanorods and substituting CTAB with sulfhydryl-terminated molecules is the most powerful strategy to decrease their toxicity [99]. Several research groups focused on the photothermal potential of gold nanorods for cancer treatment; for example, a study demonstrated that NIR-excitable Au@PEG nanorods irradiated with a 793 nm laser with a safe power density of 1 W/cm^2^ exhibited effective photothermal conversion capacity, converting NIR light into local heat of up to 53.3 °C in 9 min. Furthermore, AuNRs@PEG have shown great chemical stability and outstanding biocompatibility for cervical cancer via in vitro and in vivo studies of a hemolysis test, blood routine assays, and histological examinations [100]. Gold nanorods can also be modified by bovine serum albumin (BSA) to reduce their cytotoxicity; a recent investigation (Fig. 6(a–e)) showed that AuNRs with an absorption peak in the second NIR window (NIR-II) (1000 nm–1300nm) coated with BSA could effectively treat breast cancer. The in vitro study included the irradiation of AuNRs@BSA with a 1064 nm laser with a power density of 0.75 W/cm^2^, increasing temperature to 85.5 °C. These results confirmed that AuNRs@BSA possesses excellent absorption in the NIR-II, high photothermal conversion efficiency, and good photostability, which pushed the researchers to pursue in vivo studies [38]. In another study, gold nanorods were encased in albumin nanoparticles to exploit biocompatible albumin as a carrier to enhance tumor targetability and in vivo photothermal activity. The photothermal conversion efficiency was evaluated in vitro by irradiating the AuNRs-Alb-NPs with an 808 nm laser at 1.5 W/cm^2^ for 5 min; the temperature increased from 26 °C to 61 °C and in vivo by injecting the same nanoparticles into tumor-bearing mice and irradiating under the same conditions; the tumor volume decreased [101]. Another study highlighted the effectiveness of two-size gold nanorods in promoting cell death through photothermal therapy in glioblastoma and melanoma cell lines. This study irradiated cells with an 808 nm laser at 4.5 W for 10 min. This led to nearly complete efficacy in eliminating glioblastoma cells and melanoma cells, even at a physiological temperature of 37 °C. These findings underscore the potential of size-optimized GNRs as robust agents for targeted cancer therapy [102]. Building on the promising results of size-optimized AuNRs in glioblastoma and melanoma PTT, another study demonstrated the therapeutic potential of polyvinylpyrrolidone (PVP)--capped AuNRs combined with near-infrared laser irradiation for the treatment of aggressive mammary tumors. The study employed an 808 nm NIR continuous-wave diode laser at 200 mW/cm^2^ for 5 min, resulting in significant tumor degeneration and modulation of immune responses against highly invasive rat mammary carcinoma in vivo. These findings suggest that PVP-capped AuNRs, coupled with NIR laser irradiation, could offer a promising alternative to traditional chemotherapy for breast cancers, particularly in recurrent and early-stage disease variants [103]Another study underscored the importance of AuNRs size in optimizing treatment efficiency. Smaller-sized GNRs not only demonstrated higher cellular uptake but also required lower laser energy and achieved faster cell death compared to larger rods, with energies as low as 28 pJ for AuNR-1 in 90 min. Furthermore, simulation analyses indicated that smaller AuNRs formed more compact clusters, enhancing electromagnetic coupling and broadening absorption spectra for flexible light source usage. Collectively, these studies emphasize the potential of tailoring AuNRs size and surface modifications to enhance therapeutic outcomes across diverse cancer models [104]. Gold nanorods have been shown to be effective in targeting and destroying cancer cells over other nanomaterials in vitro studies and in vivo tests for a variety of cancer cells, however, the toxicity of the surfactant used for their synthesis is a matter of concern.

### 4.3 Au nanoshells

Gold nanoshells comprise a spherical silica core encased in a thin gold metallic shell. Their core-shell structure enables them to be promising candidates for photothermal therapy. By varying the core-to-shell ratio, the plasmon resonance of gold nanoshells can be tuned from the visible to the NIR region [105]. Gold nanoshells were first synthesized by Oldenburg et al. [96] using the seeded growth method, and this process has been extensively used over the previous decades. However, it was demonstrated that this method is complex and time-consuming, which pushes researchers to investigate more and develop or modify this process to synthesize gold nanoshells. For example, Brito-Silva et al. [106] have modified the standard seeded growth process to reduce the overall time of the synthesis and improve the reproducibility and size distribution of the nanoshells. Briefly, the synthesis of AuNShs consists of first synthesizing silica nanoparticles by reducing the silica precursor Tetraethyl orthosilicate (TEOS) with ammonium hydroxide.

The organosilane molecules (3-aminopropyl trimethoxysilane) are then adsorbed into the silica nanoparticles. After that, small gold nanoparticles (1–2 nm) are added and bonded covalently to the organosilicane@SiNPs via the amine group. Gold-decorated silica nanoparticles are used as nucleation sites to reduce chloroauric acid and potassium carbonate by a sodium borohydride solution, which increases gold coverage on the nanoparticle surface. As a photothermal agent, gold nanoshells can transform absorbed NIR light into heat and destroy the targeted cancer cells. Many research studies have been done to evaluate the photothermal potential of AuNShs; for example, melanoma cancer cells were treated with glycosylated gold nanoshells (G-AuNShs) and exposed to an 808 nm NIR laser with a power of 0.9 W/cm^2^. The gold nanoshells were synthesized and functionalized with glucosamine to enhance the cancer cell's permeability and retention of the gold nanoparticles. The results showed that the viability of cancer cells incubated with G-AuNShs decreased after exposure to NIR light, proving that glycosylated gold nanoshells can efficiently induce localized photothermal damage to cancer cells [107]. Other researchers studied the photothermal conversion efficiency of pegylated gold nanoshells conjugated with anti-human CD47 monoclonal antibodies to treat ovarian cancer. They first synthesized gold nanoshells and modified their surfaces with PEG-SH to facilitate their conjugation to the antibody. After that, the in vitro and in vivo experiments were carried out by incubating the nanoparticles with ovarian cancer cells and irradiating them with 808 nm at a power density of 2 W/cm^2^. The in vitro results showed that the temperature increased and reached 50 °C when treating with pegylated gold nanoshells and irradiating with NIR, as well as the in vivo study where the tumor size decreased after the nanoparticles treatment which confirmed that pegylated gold nanoshells could be an efficient photothermal agent for cancer therapy [108]. In another study, the photothermal effect of gold nanoshells was evaluated on prostate cancer cells using an 800 nm NIR laser at a power density of 4 W/cm^2^. They reported that, under NIR exposure, gold nanoshells had the best cellular uptake, were optically and thermally resistant, and could destroy cancer cells effectively, indicating that AuNShs have great photothermal potential [109]. Gold nanoshells were the first photothermal nanoparticles to undergo clinical trials [110].

Recently, a beta clinical test was conducted to efficiently treat prostate tumors using gold nanoshells mediated heat ablation combined with magnetic resonance-ultrasound fusion imaging technology. The therapy was successful in 94 %, with a safe process and no side effects [111]. Gold nanoshells have also demonstrated significant potential in the photothermal therapy of colon cancer. A study reported that AuNShs were successfully synthesized and characterized, with their photothermal capability investigated using intense pulsed light (IPL) irradiation. An IPL filter for 610–1200 nm wavelengths was employed, delivering 42.5 J/cm^2^ energy per 24 ms pulse. Applying 20 pulses in 1 min raised the media temperature to approximately 60 °C, leading to a significantly higher cancer cell ablation rate than untreated or non-irradiated controls. These findings underscore the promise of GNS-based photothermal approaches for effective colon cancer treatment [112].

### 4.4 Au nanostars

Gold nanostars or branched gold nanoparticles have great potential in photothermal therapy applications. Their multiple sharp branches act as “lightning rods” and can effectively transform light into heat. They exhibit tunable plasmon band, intense absorption in the NIR region, and a low scattering effect [113]. Gold nanostars synthesis is based on the seeded growth method. It includes first the preparation of gold seeds by reducing gold salt (HAuCl_4_) with sodium citrate in a boiled aqueous solution, then mixing the freshly prepared seeds with silver nitrate (AgNO_3_), ascorbic acid, and HAuCl_4_ for the growth of the nanoparticles [78]. This approach produces monodisperse and highly reproducible nanoparticles. Gold nanostars can have a variety of morphologies, from a basic three-branching structure to multiple sharp branches. These branches boost the electromagnetic properties of AuNSts that efficiently convert laser light into heat and make them excellent transducers for photothermal therapy applications. Several studies reported the application of AuNSts as an efficient photothermal agent for cancer treatment; for example, Pegylated gold nanostars were used in vitro and in vivo for SKBR3 breast cancer cells. It was demonstrated that photothermal ablation was observed when using AuNSts@PEG under a 785-nm continuous-wave laser at 1.1 W/cm^2^ for 10 min [114]. In another study, branched gold nanoparticles were conjugated with anti-HER2 nanobodies and evaluated as a photothermal agent in vitro. The conjugated nanoparticles were incubated with SKOV3 cells and irradiated with a 690 nm laser, and the cells were also exposed to only NIR irradiation as a control. As a result, cells irradiated with NIR light without nanoparticles showed no photodamage. However, photothermal destruction of the cells incubated with branched gold nanoparticles was observed after laser irradiation [115]. The great potential of aptamer-conjugated gold nanostars (Apt-AuNSts) in photothermal therapy was also reported. Synthesized gold nanostars were modified with aptamers, cultured with HeLa cells, and then irradiated with an 808 nm laser for 10 min at different power densities. Results from an in vitro photothermal treatment study showed that Apt-AuNSts can efficiently transmit heat to cancer cells, and flow cytometry analysis revealed that 64.6 % of HeLa cells underwent death after laser irradiation [116]. In another investigation, researchers synthesized spiky gold nanoparticles functionalized with O-(2-carboxyethyl)-O0 -(2-mercaptoethyl) heptaethylene glycol (HS-PEG7-COOH) and explored their photothermal conversion efficiency in colon cancer cells. The nanoparticles were incubated with the cancer cells line and irradiated with an 808 nm laser at 6.6 W/cm^2^ for 3min, which caused rapid temperature ramping and led to cellular death. The spiky gold nanoparticles show a high efficiency of light-to-heat conversion and remain stable even after repeated heating and cooling cycles [117]**.**

Another advancement in nanostar-based PTT involves the synthesis of multifunctional AuNSts with high yields through a seed-mediated growth method. These nanostars exhibit broad NIR light absorption and excellent photothermal conversion properties. They are suitable for dual applications in surface-enhanced Raman scattering (SERS) imaging and photothermal therapy across NIR-I and NIR-II windows. Functionalized with cancer-targeting ligands, they demonstrated efficient cellular uptake for imaging A549 cancer cells alongside promising therapeutic effects for targeted photothermal treatments [73]. Another innovative approach features AuNSts nanoplatforms functionalized with dendritic polyglycerol (dPG) and loaded with retinoic acid (RA) for selective targeting and treatment of breast cancer stem cells (CSCs). The platform's HA decoration enables multivalent interactions with CD44 receptors on CSCs, promoting effective targeting. This multifunctional system demonstrated dual therapeutic benefits by combining RA-induced differentiation of CSCs and high photothermal ablation efficacy, significantly inhibiting tumor growth and self-renewal in breast cancer models. This strategy underscores the potential of nanostar-based platforms for precision cancer therapy [118].

### 4.5 Au nanocages

**Fig. 7:**
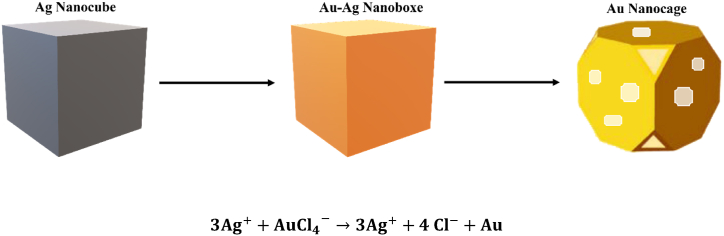
Schematic illustration of the galvanic replacement reaction

Gold nanocages are a novel class of gold nanostructures especially appealing for photothermal therapy applications. They are straightforward, can be easily and precisely tuned in the visible and NIR regions, and have compact sizes, strong absorption, and high efficiency for light-to-heat conversion [119]. Gold nanocages are synthesized by using a galvanic replacement reaction between silver nanocubes and chloroauric acid (HAuCl_4_) (Fig. 7) in an aqueous solution [120]. In a typical reaction, a solution of HAuCl_4_ is titrated into a boiling solution of Ag nanocubes. Due to the disparate electrochemical potentials of these two metals, the silver will release its electrons and dissolve into the solution as ions. At the same time, a thin layer of gold is formed on the exterior surface of the nanocube and finally generates AuNCs [121]. The absence of a surfactant stabilizer like CTAB in the synthesis of AuNCs makes them more biocompatible and non-toxic; the large and strong absorption in the NIR region allows AuNCs to be efficient transducers, and the active surface of AuNCs permits their ease of functionalization. Combining these features contributes to the widespread application of AuNCs in photothermal therapy. It was reported that gold nanocages modified with a monolayer of PEG are an efficient transducer to treat a bilateral tumor mice model. In brief, AuNCs@PEG were accumulated in the tumor and irradiated with an 808 nm continuous-wave diode laser at a power density of 0.7 W cm^−2^ for 10 min. The temperature increased rapidly to reach 55 °C within 2 min irradiation. The results demonstrated that gold nanocages functionalized with PEG have the potential to function as an effective photothermal agent for cancer treatment [122]. In another study, gold nanocages were synthesized, and their in vitro photothermal conversion was evaluated using a 750 nm laser on a breast cancer cell line. The temperature of the AuNC solution increased to 43 °C after irradiation, and the MTT assay showed that the nanocages induced a 96.41 % reduction in cell viability [123]. In a recent study, IR780, a lipophilic cationic heptamethine dye with strong optical absorption in the NIR light, was proven to be a promising candidate for enhancing the photothermal effect of AuNCs. Briefly, gold nanocages were synthesized, then stabilized with pluronic (AuNCs-Plu) to reduce their toxicity, and after that, modified with IR780 to finally form AuNCs-Plu-IR780. The photothermal efficiency was studied under 808 nm laser irradiation, and it was approved that AuNCs-Plu-IR780 can be an excellent photothermal agent for in vivo and in vitro experiments [124]. Additional research studied the photothermal therapeutic effect of AuNCs modified with Epigallocatechin gallate (EGCG). AuNCs@EGCG were successfully designed and tested under NIR irradiation. They showed an excellent photothermal response to near-infrared laser irradiation, sustained high stability after several cycles of laser irradiation, and reduced the proliferation of cancer cells by 50 % [125].

Furthermore, in another study, gold nanocages have been synthesized with surface plasmon resonance peaks tuned to 810 nm for targeted photothermal cancer therapy. Functionalized with thiolated PEG and conjugated with HER2 antibodies, these nanocages effectively target EGFR2-overexpressing SK-BR-3 breast cancer cells. Photothermal studies revealed that the nanocages achieve selective cancer cell destruction at a low power density threshold of 1.5 W/cm^2^—significantly lower than gold Nanoshells (35 W/cm^2^) and gold nanorods (10 W/cm^2^). This reduced threshold is attributed to their large absorption cross-section rather than microbubble formation. Control experiments confirmed no cell damage without nanocage treatment, highlighting their promise as potent agents for photothermal therapy [126]. Moreover, CD44-targeted PEGylated gold nanocages (CD44-PEG-GNCs) were synthesized and demonstrated enhanced cellular uptake compared to non-targeted PEG-GNCs. The combined treatment of CD44-PEG-GNCs with photothermal therapy and radiation therapy (RT) showed superior effectiveness in vitro against 4T1 cells. In vivo, intravenous administration followed by PTT and RT efficiently eradicated tumor cells with minimal damage to surrounding healthy tissue. These results suggest CD44-PEG-GNCs as a promising therapeutic platform to overcome resistance to radiation therapy with reduced side effects [127].

## 5 Factors influencing photothermal therapy efficiency

As already mentioned, plasmonic photothermal therapy is a rapidly developing therapeutic approach that utilizes the photothermal effect of gold nanoparticles to initiate localized hyperthermia in cancer cells, leading to their death. The efficiency of PPTT is influenced by various factors that impact the absorption and conversion of light energy into heat. Identifying these factors is crucial to optimize and improve the efficiency of PTT. The nanoparticle size is one of the critical elements influencing the efficiency of plasmonic photothermal therapy. In general, smaller nanoparticles exhibit higher photothermal conversion efficiency due to their large surface area-to-volume ratio [35]. Because of this, they absorb and scatter light better, which causes a higher temperature to rise in the tumor area and makes them more effective. Furthermore, the size of nanoparticles influences their biodistribution, cellular uptake, and clearance from the body. Smaller nanoparticles tend to have high penetration but are quickly cleared away, whereas large-sized nanoparticles have a longer retention time but cannot penetrate deeply into tumors [128]. Besides, the Laser provides the energy needed to heat the nanoparticles, which in turn causes hyperthermia that damages the cancer cells. In this context, several laser parameters can impact the efficiency of PTT, including the wavelength, power density, pulse duration, and repetition rate [129]. The laser wavelength should be selected to match the localized surface plasmon resonance of the nanoparticles to maximize their absorption and heating efficiency. Moreover, the power density of the Laser refers to the amount of energy delivered to the nanoparticles; it needs to be carefully controlled to avoid damaging the healthy tissues surrounding the tumor [130]. In general, the power density used for photothermal therapy ranges from 0.1 to 10 W/cm^2^, depending on the type of nanoparticle, laser wavelength, and the depth of the tumor (Table 3). The effectiveness of PTT is also affected by the pulse duration and repetition rate of the Laser [131]. The pulse duration is a critical parameter determining the amount of heat the nanoparticles generate. The repetition rate plays a crucial role in PTT efficiency; for example, a higher repetition rate can generate more heat. However, excessively high repetition rates can cause overheating. In other words, these two critical parameters must be carefully optimized to ensure that the heat generated by the nanoparticles is sufficient to destroy the cancer cell without any side effects.

**Table 3:** Photothermal conditions of different gold nanostructures.

Nanoparticle	Size(nm)	Laser wavelength (nm)	Power density (W/cm^2^)	Reference
Nanospheres	15–20	808	0.3	[78]
Nanospheres	5 15 19 40 50	532	0.22	[92]
Nanospheres	20	808	2.5	[89]
Nanospheres	95	808	1	[132]
Nanospheres	14	532	0.25	[94]
Nanorods	40	808	0.3	[78]
Nanorods	89.24x8.64	1064	0.75	[38]
Nanorods	20.5x4.6	808	1.5	[101]
Nanorods	36.2x9.1	808	0.8	[133]
Nanorods	68x20 40x10	808	4.5	[102]
Nanorods	16x5.3	808	0.2	[103]
Nanorods	54.6x13.2	808	1	[134]
Nanorods	50.8x15.1 53.9x15.2 79.9x19.1 73.1x13.4	742 776 874 930	–	[104]
Nanorods	45x10	800	1.6	[135]
Nanostars	50	808	0.3	[78]
Nanostars	180	808	6.6	[117]
Nanostars	200	660	0.5	[136]
Nanostars	220	785	0.39	[73]
Nanostars	39.19	808	2	[137]
Nanostars	40	808	1	[118]
Nanostars	30	980	0.8	[138]
Nanoshell	40	808	0.9	[107]
Nanoshell	40	800	4	[109]
Nanoshell	73	808	0.9	[139]
Nanoshell	3.7	610–1200 nm	–	[112]
Nanocage	47.4x37.1x5.2	808	0.8	[133]
Nanocage	48	808	0.7	[122]
Nanocage	45	810	1.5	[126]
Nanocage	–	850	1	[140]
Nanocage	58.14	808	2.5	[127]

This review underscores the significant potential of gold nanoparticles as highly promising photothermal agents for cancer treatment due to their unique optical properties, including tunable surface plasmon resonance and selective heat generation. These features allow precise tumor targeting and effective ablation while minimizing damage to surrounding healthy tissues, positioning them as ideal candidates for clinical applications. Preclinical studies have demonstrated their efficacy in eradicating tumors with minimal side effects [111], and early-phase clinical trials, such as AuroLase therapy utilizing silica-gold nanoshells (AuNSs), are currently underway to evaluate their safety and effectiveness in human patients. AuroLase therapy, developed by Nanospectra Biosciences, Inc., targets various cancers, including localized prostate cancer and metastatic lung tumors, marking a significant step toward clinical adoption [17]. The transition of AuNP-assisted photothermal therapy from preclinical to clinical stages represents a critical milestone in establishing its real-world medical viability. Preclinical studies in xenograft models and spontaneous tumors in animals have provided strong evidence of safety and efficacy, forming a robust foundation for further clinical exploration [17]. However, translating PPTT into routine clinical practice necessitates addressing several challenges, including long-term toxicity, biodistribution, pharmacokinetics, scalability of production, and regulatory approval [141]. This highlights the critical need to standardize treatment conditions, including nanoparticle types, laser intensities, and cancer models, to enable cross-study comparisons and streamline clinical adoption. Furthermore, strategies like surface functionalization to enhance biocompatibility, cost-effective synthesis methods, and rigorous clinical validation studies are essential for advancing AuNP-based therapies. Future directions include developing intelligent AuNP-assisted PPTT systems and exploring their integration with other therapeutic modalities, such as immunotherapy and surgery, to enhance treatment efficacy and broaden applications. In conclusion, AuNP-assisted PPTT holds substantial potential as a transformative cancer treatment modality. The ongoing clinical trials, preclinical successes, and a focus on addressing critical challenges underscore the significant progress toward realizing its clinical application, promising a new frontier in precision cancer therapy.

## 6 Challenges and limitations of gold nanoparticles in photothermal therapy compared to conventional cancer treatments

Despite the potential of AuNPs in PTT, several limitations must be addressed to optimize their application and ensure their safety and efficacy in clinical settings.

• **Size and Clearance Issues:** The size of AuNPs plays a pivotal role in their biodistribution, clearance, and therapeutic effectiveness. Nanoparticles smaller than 8 nm are typically excreted through the renal pathway, while those larger than 200 nm are sequestered by the reticuloendothelial system (RES) and accumulate in the liver and spleen [142]. This size-dependent clearance limits the ability of smaller AuNPs to remain in circulation long enough to reach tumor sites, reducing their therapeutic efficiency. Conversely, larger particles risk prolonged retention in non-target organs, which can lead to toxicity concerns over time. Balancing the size of AuNPs to maximize tumor accumulation while minimizing off-target effects remains a significant challenge.
• **Cytotoxicity and Surface Reactivity:** The physicochemical properties of AuNPs, particularly their size, charge, and surface reactivity, influence their cytotoxic potential. For instance, cationic AuNPs are more likely to interact with negatively charged cellular membranes, leading to hemolysis and inflammatory responses [142]. Additionally, AuNPs with high surface reactivity may generate oxidative stress, damaging healthy cells and tissues. As mentioned before, surface functionalization strategies, such as coating AuNPs with PEG, can mitigate these effects [143].
• **Biocompatibility Challenges:** The biocompatibility of AuNPs is influenced by factors such as zeta potential, hydrophobicity, and colloidal stability. Poorly dispersed or highly reactive particles are prone to aggregation, reducing their therapeutic efficacy and increasing toxicity risks [142].
• **Variability in Tumor Targeting:** AuNPs rely on the enhanced permeation and retention (EPR) effect for passive accumulation in tumor tissues. However, the efficiency of this mechanism varies based on the tumor's microenvironment, vascular density, and interstitial pressure [144]. The presence of monocyte-phagocytic cells further reduces the specificity of GNP delivery to cancer cells. While surface functionalization with ligands, antibodies, or peptides can improve targeting specificity, variations in tumor biology across patients and cancer types complicate achieving uniform therapeutic outcomes.
• **Surface Modifications and Biological Interactions:** Modifying the surface of AuNPs to improve targeting or stability often introduces new challenges. Nanoscale surface roughness, ligand density, and functional group arrangement influence the interactions between AuNPs and biological systems. These factors affect cellular adhesion, internalization, and trafficking, potentially altering therapeutic outcomes [142]. The complexity of these interactions highlights the need for standardized surface modification protocols to ensure consistent performance.
• **Variability in Patient Responses:** Individual variability in tumor biology, immune system activity, and overall health significantly impacts the efficacy of GNP-based PTT treatments. Factors such as the heterogeneity of tumor vasculature and immune cell activity can lead to inconsistent biodistribution and therapeutic outcomes [145]. To address this variability and optimize clinical results, personalized approaches to nanoparticle design and treatment planning are necessary.
• **Retention and Long-Term Safety Concerns:** AuNPs, particularly those with larger sizes or non-biodegradable coatings, can persist in tissues for extended periods. Prolonged retention raises concerns about bioaccumulation and potential long-term toxicity [146]. While surface modifications, such as PEGylation, can increase circulation time, they may also hinder renal clearance, resulting in accumulation in organs like the liver and spleen [142]. Long-term studies are essential to understand the excretion patterns, biodegradation, and chronic effects of AuNPs on organ function and systemic health.
• **Trade-offs between Efficacy and Distribution:** The therapeutic efficacy of AuNPs-based PTT must be carefully balanced against risks associated with non-specific distribution. While AuNPs enhance localized photothermal effects at tumor sites, their unintended accumulation in healthy tissues poses challenges. This necessitates precise control over nanoparticle design, functionalization, and dosing strategies to minimize off-target effects and maximize therapeutic benefits.
• **Challenges in Clinical Translation**: Scaling up the synthesis of AuNPs for clinical applications while maintaining consistency in size, shape, and surface properties presents a significant challenge [147]. Small parameter variations can alter biodistribution, therapeutic efficacy, and safety profiles. Regulatory approval processes require stringent quality control measures, complicating the clinical translation of GNP-based PTT [145].

Addressing these limitations requires a multidisciplinary approach integrating materials science, toxicology, and pharmacology advancements. Innovations in nanoparticle engineering, such as developing biodegradable coatings and active targeting mechanisms coupled with robust preclinical evaluations, are critical to overcoming these barriers. By addressing these challenges, AuNPs can fulfill their potential as a powerful tool in cancer photothermal therapy.

To provide a comprehensive understanding of the therapeutic potential of photothermal therapy compared to conventional cancer treatments, Table 4 summarizes key aspects such as effectiveness, advantages, and disadvantages [148].

**Table 4:** Comparison of photothermal therapy and conventional cancer treatments, emphasizing their respective advantages, disadvantages, and effectiveness.

Treatment	Advantages	Disadvantages	Effectiveness	Reference
Photothermal therapy	Targeted treatment Reduced side effect Biocompatibility Versatility	Limited to localized Tumor Biological fate of AuNPs Inconsistencies in treatment Light penetration limitations Long-term effects (Unknown)	Highly effective for localized tumors, with precise control overheat-induced cell death	[17,[149], [150], [151]]
Chemotherapy	Wide Applicability Established Protocols Combination Potential with other therapies	Systemic toxicity Drug resistance Non-specific targeting Side effects Management	Effective against both primary tumors and metastatic cancer cells due to systemic action	[9,152,153]
Radiotherapy	Targeted tumor destruction Non-invasive (external beam) Wide applicability Combination with other therapies	Side effects Long-term effects Limited to localized treatment Resistance to radiation Cumulative dose limitation Impact on healthy cells	High local control rates and enhance the effectiveness of other therapies	[11,154,155]
Surgery	Definitive tumor removal Diagnostic and staging tool Used in combination with other therapies (e.g., chemotherapy and radiotherapy	Invasiveness Limited to localized disease Incomplete resection recovery time	High efficacy for localized tumors, especially in the early stages	[[156], [157], [158]]

## 7 Conclusion

Gold nanoparticles have emerged as promising photothermal agents for cancer treatment. The unique optical properties of gold nanoparticles, biocompatibility, and ease of surface functionalization make them ideal candidates for targeted photothermal therapy. Various studies have demonstrated that gold nanoparticles can efficiently convert light energy into heat, leading to the selective destruction of cancer cells. Furthermore, the surface modification of gold nanoparticles with targeting ligands can improve their tumor specificity and enhance their therapeutic efficacy. In this paper, we spotlight gold nanoparticles' diverse and unique properties, including their high surface area-to-volume ratio, which provides a large surface area for functionalization. Their unique localized surface plasmon resonance also enables efficient light absorption and conversion into heat. Moreover, their excellent biocompatibility and inertness toward biological systems make them suitable for photothermal therapy applications. We also presented an overview of the different types of surface modification techniques, such as physisorption and chemisorption. We then reviewed the application of different shapes of gold nanoparticles in the photothermal therapy of cancer. Among various shapes, nanospheres, nanorods, nanoshells, nanostars, and nanocages have been widely investigated due to their distinct features. For example, gold nanospheres are easy to synthesize and functionalize. Gold nanorods and gold nanocages exhibit strong absorption in the NIR region, so they may effectively convert light into heat through the LSPR effect. Gold nanostars are distinguished by their large surface area owing to their multiple branches. Gold nanoshells have a core-shell structure that fine-tunes light absorption ranging from the visible to the NIR region. Despite the promising advancements, the diverse shapes of gold nanoparticles present both unique advantages and inherent limitations, necessitating further comparative research to identify the most suitable morphology for photothermal therapy applications. This research must also address long-term toxicity concerns, which remain a significant barrier to commercializing AuNPs as a cancer treatment. While AuNPs possess exceptional optical properties, biocompatibility, and multifunctionality, their transition from laboratory studies to clinical and commercial implementation requires overcoming numerous challenges. A primary obstacle is the development of scalable and reproducible methods for synthesizing AuNPs with consistent size, shape, and functionalization factors essential for clinical translation and large-scale production. Additionally, the long-term safety profile of AuNPs, including their toxicity, stability, and biodistribution, must be rigorously assessed through comprehensive and standardized clinical trials to ensure patient safety and meet regulatory requirements. Economic challenges further block commercialization, as the high nanoparticle synthesis and functionalization costs must be addressed by devising more efficient and cost-effective production strategies. Enhancing the storage stability of AuNPs is also critical to improving their economic feasibility and widespread adoption. To fully realize the potential of AuNPs in PTT, it is imperative to ensure precise targeting, optimize nanoparticle properties to maximize therapeutic efficacy, and navigate complex regulatory pathways. Addressing these challenges will facilitate the clinical application of AuNPs and establish them as a transformative approach in cancer therapy, bridging the gap between innovative laboratory findings and practical healthcare solutions.

Overall, the application of gold nanoparticles as photothermal agents has shown great potential for cancer treatment and could pave the way for developing new and more effective therapeutic strategies in the future.

## CRediT authorship contribution statement

**Amina Badir:** Writing – original draft. **Siham Refki:** Writing – original draft, Writing – review & editing, Supervision. **Zouheir Sekkat:** Writing – review & editing, Validation, Supervision.

## Data and code availability statement

No new data was generated for the research described in the article.

## Declaration of competing interest

The authors declare that they have no known competing financial interests or personal relationships that could have appeared to influence the work reported in this paper.

## References

[1] Sung H., Ferlay J., Siegel R.L., Laversanne M., Soerjomataram I., Jemal A., Bray F. (2021). Global cancer statistics 2020: GLOBOCAN estimates of incidence and mortality worldwide for 36 cancers in 185 countries. CA Cancer J. Clin..

[2] Bidram E., Esmaeili Y., Ranji-Burachaloo H., Al-Zaubai N., Zarrabi A., Stewart A., Dunstan D.E. (2019). A concise review on cancer treatment methods and delivery systems. J. Drug Deliv. Sci. Technol..

[3] Chuang Y.-C., Lee H.-L., Chiou J.-F., Lo L.-W. (2022). Recent advances in gold nanomaterials for photothermal therapy. J. Nanotheranostics.

[4] Tohme S., Simmons R.L., Tsung A. (2017). Surgery for cancer: a trigger for metastases. Cancer Res..

[5] Gomathi Mohan B.V., Ayisha Hamna T.P., Jijo A.J., Saradha Devi K.M., A.N. (2019). Recent advances in radiotherapy and its associated side effects in cancer—a review. J. Basic Appl. Zool..

[6] Koka K., Verma A., Dwarakanath B.S., Papineni R.V.L. (2022). Technological advancements in external beam radiation therapy (EBRT): an indispensable tool for cancer treatment. Cancer Manag. Res..

[7] Chabner B.A., Roberts T.G. (2005). Chemotherapy and the war on cancer. Nat. Rev. Cancer.

[8] Schirrmacher V. (2019). From chemotherapy to biological therapy: a review of novel concepts to reduce the side effects of systemic cancer treatment. Int. J. Oncol..

[9] Wara W.M. (1981). Pediatr. Oncol. 1 Spec. Sect. Rare Primit. Neuroectodermal Tumors.

[10] van den Boogaard W.M., Komninos D.S., Vermeij W.P. (2022). Chemotherapy side-effects: not all DNA damage is equal. Cancers.

[11] Shirzadfar H., Khanahmadi M. (2018). Current approaches and novel treatment methods for cancer and radiotherapy. Int. J. Biosens. Bioelectron..

[12] Broadbent M., Chadwick S.J., Brust M., Volk M. (2024). Gold nanoparticles for photothermal and photodynamic therapy. ACS Omega.

[13] Yu C., Xu L., Zhang Y., Timashev P.S., Huang Y., Liang X.-J. (2020). Polymer-based nanomaterials for noninvasive cancer photothermal therapy. ACS Appl. Polym. Mater..

[14] Liu Y., Bhattarai P., Dai Z., Chen X. (2019). Photothermal therapy and photoacoustic imaging via nanotheranostics in fighting cancer. Chem. Soc. Rev..

[15] Yu S., Xia G., Yang N., Yuan L., Li J., Wang Q., Li D., Ding L., Fan Z., Li J. (2024). Noble metal nanoparticle-based photothermal therapy: development and application in effective cancer therapy. Int. J. Mol. Sci..

[16] Stabile J., Najafali D., Cheema Y., Inglut C.T., Liang B.J., Vaja S., Sorrin A.J., Huang H.-C. (2020). Nanoparticles Biomed. Appl..

[17] Ali M.R., Wu Y., El-Sayed M.A. (2019). Gold-nanoparticle-assisted plasmonic photothermal therapy advances toward clinical application. J. Phys. Chem. C.

[18] Jaque D., Martínez Maestro L., Del Rosal B., Haro-Gonzalez P., Benayas A., Plaza J.L., Martín Rodríguez E., García Solé J. (2014). Nanoparticles for photothermal therapies. Nanoscale.

[19] Deng X., Guan W., Qing X., Yang W., Que Y., Tan L., Liang H., Zhang Z., Wang B., Liu X. (2020). Ultrafast low-temperature photothermal therapy activates autophagy and recovers immunity for efficient antitumor treatment. ACS Appl. Mater. Interfaces.

[20] Li Y., Miao Z., Shang Z., Cai Y., Cheng J., Xu X. (2020). A visible‐and NIR‐light responsive photothermal therapy agent by chirality‐dependent MoO3− x nanoparticles. Adv. Funct. Mater..

[21] Jabeen I., Rashid Z., Waheed R., Zafar S., Ahmad K., Arooj I., Aleem A., Batool S., Islam N., Ahmad I. (2025). Green synthesis and biological applications of Peganum harmala mediated copper oxide nanoparticles. J. Mol. Struct..

[22] Yi X., Duan Q.-Y., Wu F.-G. (2021).

[23] Desai D.A., Mehta F.A. (2020).

[24] Singh S., Maurya P.K. (2019). Nanotechnology in modern animal biotechnology: recent trends and future perspectives.

[25] Huang X., El-Sayed M.A. (2010). Gold nanoparticles: optical properties and implementations in cancer diagnosis and photothermal therapy. J. Adv. Res..

[26] Bansal S.A., Kumar V., Karimi J., Singh A.P., Kumar S. (2020). Role of gold nanoparticles in advanced biomedical applications. Nanoscale Adv..

[27] Cabral R.M., Baptista P.V. (2013). The chemistry and biology of gold nanoparticle-mediated photothermal therapy: promises and challenges. Nano Life.

[28] Huang X., Jain P.K., El-Sayed I.H., El-Sayed M.A. (2008). Plasmonic photothermal therapy (PPTT) using gold nanoparticles. Lasers Med. Sci..

[29] Chandrakala V., Aruna V., Angajala G. (2022). Review on metal nanoparticles as nanocarriers: current challenges and perspectives in drug delivery systems. Emergent Mater.

[30] Yeh Y.-C., Creran B., Rotello V.M. (2012). Gold nanoparticles: preparation, properties, and applications in bionanotechnology. Nanoscale.

[31] Tang B., Li W., Chang Y., Yuan B., Wu Y., Zhang M., Xu J., Li J., Zhang X. (2019). A supramolecular radical dimer: high‐efficiency NIR‐II photothermal conversion and therapy. Angew. Chem. Int. Ed..

[32] Sanità G., Carrese B., Lamberti A. (2020). Nanoparticle surface functionalization: how to improve biocompatibility and cellular internalization. Front. Mol. Biosci..

[33] Bai X., Wang Y., Song Z., Feng Y., Chen Y., Zhang D., Feng L. (2020). The basic properties of gold nanoparticles and their applications in tumor diagnosis and treatment. Int. J. Mol. Sci..

[34] Choi J., Yang J., Jang E., Suh J.-S., Huh Y.-M., Lee K., Haam S. (2011). Gold nanostructures as photothermal therapy agent for cancer, Anti-Cancer Agents Med. Chem. Former. Curr. Med. Chem.-Anti-Cancer Agents.

[35] Du B., Ma C., Ding G., Han X., Li D., Wang E., Wang J. (2017).

[36] Chen Y., Xianyu Y., Jiang X. (2017). Surface modification of gold nanoparticles with small molecules for biochemical analysis. Acc. Chem. Res..

[37] Yoo J., Park C., Yi G., Lee D., Koo H. (2019). Active targeting strategies using biological ligands for nanoparticle drug delivery systems. Cancers.

[38] Zhao S., Luo Y., Chang Z., Liu C., Li T., Gan L., Huang Y., Sun Q. (2021). BSA-coated gold nanorods for NIR-II photothermal therapy. Nanoscale Res. Lett..

[39] Li S., Ju Y., Zhou J., Faria M., Ang C.-S., Mitchell A.J., Zhong Q.-Z., Zheng T., Kent S.J., Caruso F. (2022). Protein precoating modulates biomolecular coronas and nanocapsule–immune cell interactions in human blood. J. Mater. Chem. B.

[40] Rezaei B., Harun A., Wu X., Iyer P.R., Mostufa S., Ciannella S., Karampelas I.H., Chalmers J., Srivastava I., Gómez‐Pastora J. (2024). Effect of polymer and cell membrane coatings on theranostic applications of nanoparticles: a review. Adv. Healthc. Mater..

[41] Soliman M.G., Pelaz B., Parak W.J., Del Pino P. (2015). Phase transfer and polymer coating methods toward improving the stability of metallic nanoparticles for biological applications. Chem. Mater..

[42] Liu B., Nguyen H., Jiang Y., Wang A., Lensch V., Sun Z., Boyer Z., Raftopoulos P., Dai Y., MacNicol P. (2024).

[43] Jo H., Ban C. (2016). Aptamer-nanoparticle complexes as powerful diagnostic and therapeutic tools. Exp. Mol. Med..

[44] Kolovskaya O.S., Zamay T.N., Belyanina I.V., Karlova E., Garanzha I., Aleksandrovsky A.S., Kirichenko A., Dubynina A.V., Sokolov A.E., Zamay G.S., Glazyrin Y.E., Zamay S., Ivanchenko T., Chanchikova N., Tokarev N., Shepelevich N., Ozerskaya A., Badrin E., Belugin K., Belkin S., Zabluda V., Gargaun A., Berezovski M.V., Kichkailo A.S. (2017). Aptamer-targeted plasmonic photothermal therapy of cancer. Mol. Ther. Nucleic Acids.

[45] Duo Y., Yang M., Du Z., Feng C., Xing C., Wu Y., Xie Z., Zhang F., Huang L., Zeng X., Chen H. (2018). CX-5461-loaded nucleolus-targeting nanoplatform for cancer therapy through induction of pro-death autophagy. Acta Biomater..

[46] Coppola G., Cennamo F., Ciccone G., Ibba M.L., Di Vito A., Esposito C.L., Catuogno S. (2024).

[47] Dhaini B., Kenzhebayeva B., Ben-Mihoub A., Gries M., Acherar S., Baros F., Thomas N., Daouk J., Schohn H., Hamieh T., Frochot C. (2021). Peptide-conjugated nanoparticles for targeted photodynamic therapy.

[48] Gandhi S., Shastri D.H. (2024). Lipid-based nanoparticles as drug delivery system for modern therapeutics. Pharm. Nanotechnol..

[49] Dinakar Y.H., Karole A., Parvez S., Jain V., Mudavath S.L. (2023). Folate receptor targeted NIR cleavable liposomal delivery system augment penetration and therapeutic efficacy in breast cancer. Biochim. Biophys. Acta BBA-Gen. Subj..

[50] El‐Sayed N., Elbadri K., Correia A., Santos H.A. (2023). Polyethylene glycol‐stabilized gold nanostars‐loaded microneedles for photothermal therapy of melanoma. Adv. Mater. Technol..

[51] Du X., Lin W., Su H. (2019). Highly efficient polyethylene glycol‐functionalised gold nanorods for photothermal ablation of hepatocellular carcinoma cells. IET Nanobiotechnol..

[52] Wang J., Zhang Y., Jin N., Mao C., Yang M. (2019). Protein-induced gold nanoparticle assembly for improving the photothermal effect in cancer therapy. ACS Appl. Mater. Interfaces.

[53] Chen C.H., Wu Y.-J., Chen J.-J. (2016). Photo-thermal therapy of bladder cancer with Anti-EGFR antibody conjugated gold nanoparticles. Front. Biosci..

[54] Qu X., Yao C., Wang J., Li Z., Zhang Z. (2012). Anti-CD30-targeted gold nanoparticles for photothermal therapy of L-428 Hodgkin's cell. Int. J. Nanomedicine.

[55] Hong E.J., Kim Y.-S., Choi D.G., Shim M.S. (2018). Cancer-targeted photothermal therapy using aptamer-conjugated gold nanoparticles. J. Ind. Eng. Chem..

[56] Kim S.-J., Park H.-B., An E.-K., Ryu D., Zhang W., Pack C.-G., Kim H., Kwak M., Im W., Ryu J.-H. (2024). Lipid-coated gold nanorods for photoimmunotherapy of primary breast cancer and the prevention of metastasis. J. Controlled Release.

[57] Rengan A.K., Jagtap M., De A., Banerjee R., Srivastava R. (2014). Multifunctional gold coated thermo-sensitive liposomes for multimodal imaging and photo-thermal therapy of breast cancer cells. Nanoscale.

[58] Neshastehriz A., Tabei M., Maleki S., Eynali S., Shakeri-Zadeh A. (2017). Photothermal therapy using folate conjugated gold nanoparticles enhances the effects of 6 MV X-ray on mouth epidermal carcinoma cells. J. Photochem. Photobiol., B.

[59] Bertel Garay L., Mendez Sanchez S.C., Martinez Ortega F. (2018). Use in vitro of gold nanoparticles functionalized with folic acid as a photothermal agent on treatment of HeLa cells. J. Mex. Chem. Soc..

[60] Zhang L., Mazouzi Y., Salmain M., Liedberg B., Boujday S. (2020). Antibody-gold nanoparticle bioconjugates for biosensors: synthesis, characterization and selected applications. Biosens. Bioelectron..

[61] Jazayeri M.H., Amani H., Pourfatollah A.A., Pazoki-Toroudi H., Sedighimoghaddam B. (2016). Various methods of gold nanoparticles (GNPs) conjugation to antibodies. Sens. Bio-Sens. Res..

[62] Hermanson G.T. (2008). Bioconjugate techniques.

[63] Zhenxi Zhang C.Y., Wang † Sijia, Xu † Hao, Wang Bo (2015). Role of 5-aminolevulinic acid_conjugated gold nanoparticles for photodynamic therapy of cancer. J. Biomed. Opt..

[64] Mokoena D., George B.P., Abrahamse H. (2022). Conjugation of hypericin to gold nanoparticles for enhancement of photodynamic therapy in MCF-7 breast cancer cells. Pharmaceutics.

[65] Kumar S., Aaron J., Sokolov K. (2008). Directional conjugation of antibodies to nanoparticles for synthesis of multiplexed optical contrast agents with both delivery and targeting moieties. Nat. Protoc..

[66] Juan A., Cimas F.J., Bravo I., Pandiella A., Ocaña A., Alonso-Moreno C. (2020). An overview of antibody conjugated polymeric nanoparticles for breast cancer therapy. Pharmaceutics.

[67] Nicol J.R., Dixon D., Coulter J.A. (2015). Gold nanoparticle surface functionalization: a necessary requirement in the development of novel nanotherapeutics. Nanomed.

[68] Biju V. (2014). Chemical modifications and bioconjugate reactions of nanomaterials for sensing, imaging, drug delivery and therapy. Chem. Soc. Rev..

[69] Lin X., O'Reilly Beringhs A., Lu X. (2021). Applications of nanoparticle-antibody conjugates in immunoassays and tumor imaging. AAPS J..

[70] Huang P., Bao L., Zhang C., Lin J., Luo T., Yang D., He M., Li Z., Gao G., Gao B., Fu S., Cui D. (2011). Folic acid-conjugated Silica-modi fi ed gold nanorods for X-ray/CT imaging-guided dual-mode radiation and photo-thermal therapy. Biomaterials.

[71] Liang S., Li C., Zhang C., Chen Y., Xu L., Bao C., Wang X., Liu G., Zhang F., Cui D. (2015). CD44v6 monoclonal antibody-conjugated gold nanostars for targeted photoacoustic imaging and plasmonic photothermal therapy of gastric cancer stem-like cells. Theranostics.

[72] Tan H., Hou N., Liu Y., Liu B., Cao W., Zheng D., Li W., Liu Y., Xu B., Wang Z., Cui D. (2020). CD133 antibody targeted delivery of gold nanostars loading IR820 and docetaxel for multimodal imaging and near-infrared photodynamic/photothermal/chemotherapy against castration resistant prostate cancer. Nanomedicine Nanotechnol. Biol. Med..

[73] Song C., Li F., Guo X., Chen W., Dong C., Zhang J., Zhang J., Wang L. (2019). Gold nanostars for cancer cell-targeted SERS-imaging and NIR light-triggered plasmonic photothermal therapy (PPTT) in the first and second biological windows. J. Mater. Chem. B.

[74] Zhang L., Mazouzi Y., Salmain M., Liedberg B., Boujday S. (2020). Antibody-gold nanoparticle bioconjugates for biosensors: synthesis, characterization and selected applications. Biosens. Bioelectron..

[75] Kumari S., Sharma N., Sahi S.V. (2021). Advances in cancer therapeutics: conventional thermal therapy to nanotechnology-based photothermal therapy. Pharmaceutics.

[76] Ren Y., Yan Y., Qi H. (2022). Photothermal conversion and transfer in photothermal therapy: from macroscale to nanoscale. Adv. Colloid Interface Sci..

[77] Spyratou E., Makropoulou M., Efstathopoulos E.P., Georgakilas A.G., Sihver L. (2017). Recent advances in cancer therapy based on dual mode gold nanoparticles. Cancers.

[78] Yang W., Xia B., Wang L., Ma S., Liang H., Wang D., Huang J. (2021). Shape effects of gold nanoparticles in photothermal cancer therapy. Mater. Today Sustain..

[79] Georgeous J., AlSawaftah N., Abuwatfa W.H., Husseini G.A. (2024). Review of gold nanoparticles: synthesis, properties, shapes, cellular uptake, targeting, release mechanisms and applications in drug delivery and therapy. Pharmaceutics.

[80] Aguilar F., Li X., Kociak M., Campos A. (2022).

[81] Liao S., Yue W., Cai S., Tang Q., Lu W., Huang L., Qi T., Liao J. (2021). Improvement of gold nanorods in photothermal therapy: recent progress and perspective. Front. Pharmacol..

[82] Renero‐Lecuna C., Pulagam K.R., Uribe K.B., Vázquez‐Aristizabal P., Gómez‐Vallejo V., Liz‐Marzán L.M., Llop J., Henriksen‐Lacey M. (2024). Positron emission tomography‐assisted photothermal therapy with gold nanorods. Part. Part. Syst. Charact..

[83] Ngo N.M., Tran H.-V., Lee T.R. (2022). Plasmonic nanostars: systematic review of their synthesis and applications. ACS Appl. Nano Mater..

[84] Singh P., Roy S., Sanpui P., Banerjee A., Jaiswal A. (2019). Gold nanostructures for photothermal therapy. Nanotechnol. Mod. Anim. Biotechnol. Recent Trends Future Perspect..

[85] Huang J., Han Y. (2015). Recent Prog. Colloid Surf. Chem. Biol. Appl..

[86] Huang X., Qian W., El‐Sayed I.H., El‐Sayed M.A. (2007). The potential use of the enhanced nonlinear properties of gold nanospheres in photothermal cancer therapy. Lasers Surg. Med. Off. J. Am. Soc. Laser Med. Surg..

[87] Wang Y.-C., Rhéaume É., Lesage F., Kakkar A. (2018). Synthetic methodologies to gold nanoshells: an overview. Molecules.

[88] Xie X., Liao J., Shao X., Li Q., Lin Y. (2017). The effect of shape on cellular uptake of gold nanoparticles in the forms of stars, rods, and triangles. Sci. Rep..

[89] Pornnoppadol G., Cho S., Yu J.H., Kim S.-H., Nam Y.S. (2024). Cancer-targeting gold-decorated melanin nanoparticles for in vivo near-infrared photothermal therapy. Mol. Syst. Des. Eng..

[90] Turkevich P.C., Stevenson J. (1951). A study of the nucleation and growth processes I N the synthesis of, discuss. Faraday Soc..

[91] Poon W., Zhang Y.N., Ouyang B., Kingston B.R., Wu J.L.Y., Wilhelm S., Chan W.C.W. (2019). Elimination pathways of nanoparticles. ACS Nano.

[92] Jiang K., Smith D.A., Pinchuk A. (2013). Size-dependent photothermal conversion efficiencies of plasmonically heated gold nanoparticles. J. Phys. Chem. C.

[93] El-Sayed I.H., Huang X., El-Sayed M.A. (2006). Selective laser photo-thermal therapy of epithelial carcinoma using anti-EGFR antibody conjugated gold nanoparticles. Cancer Lett..

[94] Faid A.H., Shouman S.A., Badr Y.A., Sharaky M. (2022). Enhanced photothermal heating and combination therapy of gold nanoparticles on a breast cell model. BMC Chem..

[95] Amornkitbamrung L., Kim J., Roh Y., Chun S.H., Yuk J.S., Shin S.W., Kim B.W., Oh B.K., Um S.H. (2018). Cationic surfactant-induced formation of uniform gold nanoparticle clusters with high efficiency of photothermal conversion under near-infrared irradiation. Langmuir.

[96] Nasseri B., Turk M., Kosemehmetoglu K., Kaya M., Piskin E., Rabiee N., Webster T.J. (2020). The pimpled gold nanosphere: a superior candidate for plasmonic photothermal therapy. Int. J. Nanomedicine.

[97] Zhou R., Zhang M., Xi J., Li J., Ma R., Ren L., Bai Z., Qi K., Li X. (2022). Gold nanorods-based photothermal therapy: interactions between biostructure, nanomaterial, and near-infrared irradiation. Nanoscale Res. Lett..

[98] Chen H., Shao L., Li Q., Wang J. (2013). Gold nanorods and their plasmonic properties. Chem. Soc. Rev..

[99] Khan N.U., Lin J., Younas M.R., Liu X., Shen L. (2021). Synthesis of gold nanorods and their performance in the field of cancer cell imaging and photothermal therapy. Cancer Nanotechnol.

[100] Zhang L., Chen S., Ma R., Zhu L., Yan T., Alimu G., Du Z., Alifu N., Zhang X. (2021). NIR-excitable PEG-modified Au nanorods for photothermal therapy of cervical cancer. ACS Appl. Nano Mater..

[101] Seo B., Lim K., Kim S.S., Oh K.T., Lee E.S., Choi H.G., Shin B.S., Youn Y.S. (2019). Small gold nanorods-loaded hybrid albumin nanoparticles with high photothermal efficacy for tumor ablation. Colloids Surf. B Biointerfaces.

[102] Domingo-Diez J., Souiade L., Manzaneda-González V., Sánchez-Díez M., Megias D., Guerrero-Martínez A., Ramírez-Castillejo C., Serrano-Olmedo J., Ramos-Gómez M. (2023). Effectiveness of gold nanorods of different sizes in photothermal therapy to eliminate melanoma and glioblastoma cells. Int. J. Mol. Sci..

[103] Gamal H., Tawfik W., El-Sayyad H.I., Emam A.N., Fahmy H.M., El-Ghaweet H.A. (2024). A new vision of photothermal therapy assisted with gold nanorods for the treatment of mammary cancers in adult female rats. Nanoscale Adv..

[104] Zhou W., Yao Y., Qin H., Xing X., Li Z., Ouyang M., Fan H. (2024). Size dependence of gold nanorods for efficient and rapid photothermal therapy. Int. J. Mol. Sci..

[105] Gao Y., Gu J., Li L., Zhao W., Li Y. (2016). Synthesis of gold nanoshells through improved seed-mediated growth approach: brust-like, in situ seed formation. Langmuir.

[106] Brito-Silva A.M., Sobral-Filho R.G., Barbosa-Silva R., De Araújo C.B., Galembeck A., Brolo A.G. (2013). Improved synthesis of gold and silver nanoshells. Langmuir.

[107] Nouri S., Mohammadi E., Mehravi B., Majidi F., Ashtari K., Neshasteh-Riz A., Einali S. (2019). NIR triggered glycosylated gold nanoshell as a photothermal agent on melanoma cancer cells, Artif. Cells Nanomedicine Biotechnol.

[108] Wu C.C., Yang Y.C., Hsu Y.T., Wu T.C., Hung C.F., Huang J.T., Chang C.L. (2015). Nanoparticle-induced intraperitoneal hyperthermia and targeted photoablation in treating ovarian cancer. Oncotarget.

[109] Leung J.P., Wu S., Chou K.C., Signorell R. (2013). Investigation of sub-100 nm gold nanoparticles for laser-induced thermotherapy of cancer. Nanomaterials.

[110] Lv Z., He S., Wang Y., Zhu X. (2021). Noble metal nanomaterials for NIR-triggered photothermal therapy in cancer. Adv. Healthc. Mater..

[111] Rastinehad A.R., Anastos H., Wajswol E., Winoker J.S., Sfakianos J.P., Doppalapudi S.K., Carrick M.R., Knauer C.J., Taouli B., Lewis S.C., Tewari A.K., Schwartz J.A., Canfield S.E., George A.K., West J.L., Halas N.J. (2019). Gold nanoshell-localized photothermal ablation of prostate tumors in a clinical pilot device study. Proc. Natl. Acad. Sci. U. S. A..

[112] Koohi S.R., Derakhshan M.A., Faridani F., Muhammad Nejad S., Amanpour S., Tajerian R., Yarmahmoodi M., Faridi‐Majidi R. (2018). Plasmonic photothermal therapy of colon cancer cells utilising gold nanoshells: an in vitro study. IET Nanobiotechnol..

[113] Ahmad R., Fu J., He N., Li S. (2016). Advanced gold nanomaterials for photothermal therapy of cancer. J. Nanosci. Nanotechnol..

[114] Yuan H., Khoury C.G., Wilson C.M., Grant G.A., Bennett A.J., Vo-Dinh T. (2012). In vivo particle tracking and photothermal ablation using plasmon-resonant gold nanostars. Nanomedicine Nanotechnol. Biol. Med..

[115] Van De Broek B., Devoogdt N., Dhollander A., Gijs H.L., Jans K., Lagae L., Muyldermans S., Maes G., Borghs G. (2011). Specific cell targeting with nanobody conjugated branched gold nanoparticles for photothermal therapy. ACS Nano.

[116] Li Y., Wang X., Gao L., Hu P., Jiang L., Ren T., Fu R., Yang D., Jiang X. (2018). Aptamer-conjugated gold nanostars for targeted cancer photothermal therapy. J. Mater. Sci..

[117] Costantini P.E., Di Giosia M., Ulfo L., Petrosino A., Saporetti R., Fimognari C., Pompa P.P., Danielli A., Turrini E., Boselli L., Calvaresi M. (2021). Spiky gold nanoparticles for the photothermal eradication of colon cancer cells. Nanomaterials.

[118] Pan Y., Ma X., Liu C., Xing J., Zhou S., Parshad B., Schwerdtle T., Li W., Wu A., Haag R. (2021). Retinoic acid-loaded dendritic polyglycerol-conjugated gold nanostars for targeted photothermal therapy in breast cancer stem cells. ACS Nano.

[119] Cobley C.M., Au L., Chen J., Xia Y. (2010). Targeting gold nanocages to cancer cells for photothermal destruction and drug delivery. Expert Opin. Drug Deliv..

[120] Skrabalak S.E., Au L., Li X., Xia Y. (2007). Facile synthesis of Ag nanocubes and Au nanocages. Nat. Protoc..

[121] Sun Y., Xia Y. (2004). Mechanistic study on the replacement reaction between silver nanostructures and chloroauric acid in aqueous medium. J. Am. Chem. Soc..

[122] Chen J., Glaus C., Laforest R., Zhang Q., Yang M., Gidding M., Welch M.J., Xia Y. (2010). Gold nanocages as photothermal transducers for cancer treatment. Small.

[123] Rengan A.K., Kundu G., Banerjee R., Srivastava R. (2014). Gold nanocages as effective photothermal transducers in killing highly tumorigenic cancer cells, Part. Part. Syst. Charact..

[124] Hu Y., Huang S., Zhao X., Chang L., Ren X., Mei X., Chen Z. (2021). Preparation of photothermal responsive and ROS generative gold nanocages for cancer therapy. Chem. Eng. J..

[125] Gao W., Fan X., Bi Y., Zhou Z., Yuan Y. (2022). Preparation of NIR-responsive gold nanocages as efficient carrier for controlling release of EGCG in anticancer application. Front. Chem..

[126] Chen J., Wang D., Xi J., Au L., Siekkinen A., Warsen A., Li Z.-Y., Zhang H., Xia Y., Li X. (2007). Immuno gold nanocages with tailored optical properties for targeted photothermal destruction of cancer cells. Nano Lett..

[127] Zhang A., Guo W., Qi Y., Wang J., Ma X., Yu D. (2016). Synergistic effects of gold nanocages in hyperthermia and radiotherapy treatment. Nanoscale Res. Lett..

[128] Yu W., Liu R., Zhou Y., Gao H. (2020). Size-tunable strategies for a tumor targeted. Drug Deliv. Syst..

[129] Bucharskaya A., Maslyakova G., Terentyuk G., Yakunin A., Avetisyan Y., Bibikova O., Tuchina E., Khlebtsov B., Khlebtsov N. (2016).

[130] Fu S., Man Y., Jia F. (2020).

[131] Akbarzadeh F., H.N.& K.K. (2018). In vitro outlook of gold nanoparticles in photo-thermal therapy: a literature review. Lasers Med. Sci..

[132] Ghaffarlou M., Rashidzadeh H., Mohammadi A., Mousazadeh N., Barsbay M., Sharafi A., Gharbavi M., Danafar H., Javani S. (2024). Photothermal and radiotherapy with alginate-coated gold nanoparticles for breast cancer treatment. Sci. Rep..

[133] Wang Y., Black K.C.L., Luehmann H., Li W., Zhang Y., Cai X., Wan D., Al W.E.T. (2013).

[134] Navarro-Palomares E., García-Hevia L., Galán-Vidal J., Gandarillas A., García-Reija F., Sánchez-Iglesias A., Liz-Marzán L.M., Valiente R., Fanarraga M.L. (2022). Shiga toxin-B targeted gold nanorods for local photothermal treatment in oral cancer clinical samples. Int. J. Nanomedicine.

[135] Manuchehrabadi N., Toughiri R., Bieberich C., Cai H., Attaluri A., Edziah R., Lalanne E., Johnson A.M., Ma R., Zhu L. (2013). Treatment efficacy of laser photothermal therapy using gold nanorods. Int. J. Biomed. Eng. Technol..

[136] Sato K., Ogawa K., Tagami T., Ozeki T. (2024). Photothermal therapeutic effect by gold nanostars/extracellular vesicles nanocomplex on melanoma cells. J. Pharm. Sci..

[137] Jo H., Youn H., Lee S., Ban C. (2014). Ultra-effective photothermal therapy for prostate cancer cells using dual aptamer-modified gold nanostars. J. Mater. Chem. B.

[138] Liu Y., Ashton J., Moding E., Yuan H., Register J., Fales A., Choi J., Whitley M., Zhao X., Qi Y. (2015).

[139] Journal A.I., Majidi F.S., Mohammadi E., Mehravi B., Nouri S., Ashtari K., Neshasteh-riz A. (2019). Investigating the effect of near infrared photo thermal therapy folic acid conjugated gold nano shell on melanoma cancer cell line A375, Artif. Cells Nanomedicine Biotechnol..

[140] Piao J.-G., Gao F., Li Y., Yu L., Liu D., Tan Z.-B., Xiong Y., Yang L., You Y.-Z. (2018). pH-sensitive zwitterionic coating of gold nanocages improves tumor targeting and photothermal treatment efficacy. Nano Res..

[141] Zhang R., Kiessling F., Lammers T., Pallares R.M. (2023). Clinical translation of gold nanoparticles. Drug Deliv. Transl. Res..

[142] Nel A.E., Mädler L., Velegol D., Xia T., Hoek E.M., Somasundaran P., Klaessig F., Castranova V., Thompson M. (2009). Understanding biophysicochemical interactions at the nano–bio interface. Nat. Mater..

[143] Nguyenova H.Y., Kalbacova M.H., Dendisova M., Sikorova M., Jarolimkova J., Kolska Z., Ulrychova L., Weber J., Reznickova A. (2024). Stability and biological response of PEGylated gold nanoparticles. Heliyon.

[144] Ghosh P., Han G., De M., Kim C.K., Rotello V.M. (2008). Gold nanoparticles in delivery applications. Adv. Drug Deliv. Rev..

[145] Dykman L., Khlebtsov N. (2012). Gold nanoparticles in biomedical applications: recent advances and perspectives. Chem. Soc. Rev..

[146] Arif M., Nawaz A.F., Mueen H., Rashid F., Hemeg H.A., Rauf A. (2023). Nanotechnology-based radiation therapy to cure cancer and the challenges in its clinical applications. Heliyon.

[147] Dastgheib Z.S., Abolmaali S.S., Farahavar G., Salmanpour M., Tamaddon A.M. (2024). Gold nanostructures in melanoma: advances in treatment, diagnosis, and theranostic applications. Heliyon.

[148] Fernandes D.A. (2023). Liposomes for cancer theranostics. Pharmaceutics.

[149] Mackey M.A., Ali M.R., Austin L.A., Near R.D., El-Sayed M.A. (2014). The most effective gold nanorod size for plasmonic photothermal therapy: theory and in vitro experiments. J. Phys. Chem. B.

[150] Li J., Xu S. (2024). Research progress on photothermal therapy of malignant tumor cells. Appl. Comput. Eng..

[151] Deng X., Shao Z., Zhao Y. (2021). Solutions to the drawbacks of photothermal and photodynamic cancer therapy. Adv. Sci..

[152] Zhang L., Zhang Y., Xue Y., Wu Y., Wang Q., Xue L., Su Z., Zhang C. (2019). Transforming weakness into strength: photothermal‐therapy‐induced inflammation enhanced cytopharmaceutical chemotherapy as a combination anticancer treatment. Adv. Mater..

[153] Gorey K.M., Haji-Jama S., Bartfay E., Luginaah I.N., Wright F.C., Kanjeekal S.M. (2014). Lack of access to chemotherapy for colon cancer: multiplicative disadvantage of being extremely poor, inadequately insured and African American. BMC Health Serv. Res..

[154] König J., van Ewijk R., Kuhr K., Schmidberger H., Wöckel A., Kreienberg R., Blettner M. (2016). Radiotherapy effects on early breast cancer survival in observational and randomized studies: a systematic analysis of advantages, disadvantages and differences between the two study types. Breast Cancer.

[155] Chen H.H., Kuo M.T. (2017). Improving radiotherapy in cancer treatment: promises and challenges. Oncotarget.

[156] Boger M.S., Perrier N.D. (2004). Advantages and disadvantages of surgical therapy and optimal extent of thyroidectomy for the treatment of hyperthyroidism. Surg. Clin..

[157] Martelli N., Serrano C., van den Brink H., Pineau J., Prognon P., Borget I., El Batti S. (2016). Advantages and disadvantages of 3-dimensional printing in surgery: a systematic review. Surgery.

[158] White W.C., Amendola F.H. (1944). The advantages and disadvantages of closed resection of the colon. Ann. Surg..

